# Synthesis and Crystal Structure of the Europium(II) Hydride Oxide Iodide Eu_5_H_2_O_2_I_4_ Showing Blue-Green Luminescence

**DOI:** 10.3390/ijms241914969

**Published:** 2023-10-07

**Authors:** Daniel Rudolph, Thomas Wylezich, Philip Netzsch, Björn Blaschkowski, Henning A. Höppe, Philippe Goldner, Nathalie Kunkel, Jean-Louis Hoslauer, Thomas Schleid

**Affiliations:** 1 Institut für Anorganische Chemie, Universität Stuttgart, Pfaffenwaldring 55, 70569 Stuttgart, Germanyblaschkowski@iac.uni-stuttgart.de (B.B.);; 2 Institut für Anorganische Chemie, Technische Universität München, Lichtenbergstrasse 4, 85747 Garching, Germany; thomas.wylezich@tum.de; 3 Institut für Physik, Universität Augsburg, Universitätsstraße 1, 86159 Augsburg, Germany; 4 Institut de Recherche de Chimie Paris, CNRS, Chimie ParisTech, PSL University, 75005 Paris, France; 5 Institut für Anorganische Chemie, Georg-August-Universität Göttingen, Tammannstrasse 4, 37077 Göttingen, Germany

**Keywords:** europium, hydrides, oxides, iodides, Eu^2+^ luminescence, crystal structures

## Abstract

As the first europium(II) hydride oxide iodide, dark red single crystals of Eu_5_H_2_O_2_I_4_ could be synthesized from oxygen-contaminated mixtures of EuH_2_ and EuI_2_. Its orthorhombic crystal structure (*a* = 1636.97(9) pm, *b* = 1369.54(8) pm, *c* = 604.36(4) pm, *Z* = 4) was determined via single-crystal X-ray diffraction in the space group *Cmcm*. Anion-centred tetrahedra [HEu_4_]^7+^ and [OEu_4_]^6+^ serve as central building blocks interconnected via common edges to infinite ribbons parallel to the *c* axis. These ribbons consist of four trans-edge connected (Eu^2+^)_4_ tetrahedra as repetition unit, two H^−^-centred ones in the inner part, and two O^2−^-centred ones representing the outer sides. They are positively charged, according to $\overset{1}{\infty }${[Eu_5_H_2_O_2_]^4+^}, to become interconnected and charge-balanced by iodide anions. Upon excitation with UV light, the compound shows blue–green luminescence with the shortest Eu^2+^ emission wavelength ever observed for a hydride derivative, peaking at 463 nm. The magnetic susceptibility of Eu_5_H_2_O_2_I_4_ follows the Curie-Weiss law down to 100 K, and exhibits a ferromagnetic ordering transition at about 10 K.

## 1. Introduction

The simultaneous coexistence of hydride and oxide anions in one and the same compound, often misleadingly referred to as “*oxyhydrides*”, seems to be astonishing at first sight. However, the hydride oxides *Ln*HO for *Ln* = La, Ce and Pr, which might be misinterpreted as “*hydroxides*” in their chemical formula when written as *Ln*OH, were first described in the early 1960s, already [1] in a cubic unit cell. Far later, neutron diffraction studies on LaHO resulted in the determination of a revised crystal structure model based on a superstructure of the fluorite type. Ionic conductivity measurements were carried out successively [2,3]. Recently, the isotypic neodymium hydride oxide NdHO [4] as well as the analogues for samarium and the lanthanoids from gadolinium to erbium with anion-disordered fluorite-type structures [5,6] were found. Next to these ternary lanthanoid(III) compounds, quaternary lithium-bearing hydride oxides with the composition Li*Ln*_2_HO_3_ (*Ln* = La − Nd) have also been found [7]. Very recently, the hydride oxide LiLa_2_HO_3_ was reinvestigated concerning its crystal structure, with a different ordering of anions and hydride-ion conductivity in solid solutions with LiSr_2_H_3_O [8,9,10,11]. In general, mixed anionic compounds like hydride oxides have garnered a lot of attention as promising new materials with regard to their optical properties like luminescence, anion conductivity, and catalytic activity, and their applicability as 2D electronic structures [12,13]. Lately, with LiEu_2_HOCl_2_, the first lanthanoid(II) hydride oxide could be prepared as a chloride derivative, and its luminescence properties were determined [14] after several mixed anionic hydride halides with Eu^2+^ as luminescence-active cations had been investigated. Compounds like the hydride fluorides EuH*_x_*F_2−*x*_ [15], KMgHF_2_:Eu^2+^ and SrH_0.5_F_1.5_:Eu^2+^ [16] should be mentioned here, as well as the hydride halides EuHCl [17], EuHBr [18], Eu_2_H_3_Cl [18] and the Eu^2+^-doped alkaline-earth metal hydride chlorides *AE*_7_H_12_Cl_2_ (*AE* = Ca and Sr) [19]. Moreover, the Tb^3+^-luminescence of a trivalent rare-earth metal cation in a doped hydride oxide (GdHO) was first observed [20]. Rare earth metal cation-doped materials are a widely reported class of materials. Because of their luminescence properties, they find applications as phosphors in phosphor-converted white light-emitting diodes [21,22,23]. An advantage of mixed anion hydride materials as phosphors is the tuning of the emission wavelength by varying the hydride content, as was shown for the mixed hydride fluorides EuH*_x_*F_2−*x*_ [17] as well as for RbMgH*_x_*F_3−*x*_ and KMgH*_x_*F_3−*x*_ [24], recently. Such systems can be used as local probes for hydrogen content. Other possible applications for rare earth metal cation-doped substances are upconversion materials and temperature sensors [25,26,27,28,29,30]. We report the successful synthesis of a further europium (II) hydride oxide halide with the composition Eu_5_H_2_O_2_I_4_, nicely reflecting the analogy between europium and the heavy alkaline earth metals, since the barium analogue Ba_5_H_2_O_2_I_4_ is already known [31].

## 2. Results and Discussion

### 2.1. Crystal Structure

**Figure 1:**
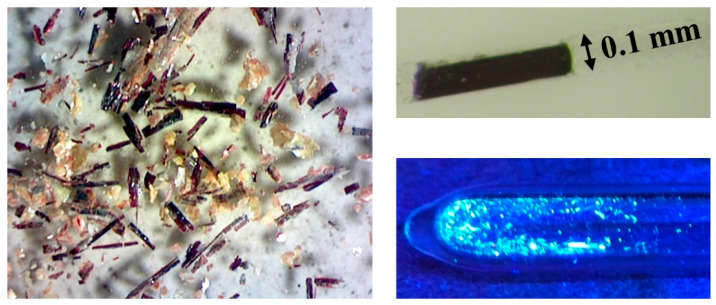
Photograph of an inhomogeneous product sample (**left**); Eu_5_H_2_O_2_I_4_: dark red, Eu_2_OI_2_: orange, Eu_4_OI_6_: yellow, NaI: white) and one single crystal of Eu_5_H_2_O_2_I_4_ (**top right**), as well as selected crystals under UV light (**bottom right**).

The europium(II) hydride oxide iodide Eu_5_H_2_O_2_I_4_ (Figure 1) crystallises isotypically to the analogous barium compound in the orthorhombic space group *Cmcm* (Table 1). Since the crystal structure showed disordered iodide anions at room temperature, a single-crystal X-ray measurement at 100 K was carried out. The disorder could not be “frozen out” into a completely ordered variant, but there were only two instead of three partially occupied positions for iodide anions needed for the description of the low-temperature disorder (Table 2). Furthermore, it was possible to refine the disordered iodide anions anisotropically, while this was not possible for the room-temperature structure. In addition, we attempted to solve the crystal structure in suitable subgroups of *Cmcm*, but the iodine disorder always remained. The presence of a merohedral twin can be excluded due to symmetry considerations, however. The dark red colour of Eu_5_H_2_O_2_I_4_ does not surprise much, since it can be observed for pure hydride halides with heavy halogens (e.g., EuHBr [18,32] and Eu_2_H_3_*X* (*X* = Br [18,33] and I [18,34]) as well.

**Figure 2:**
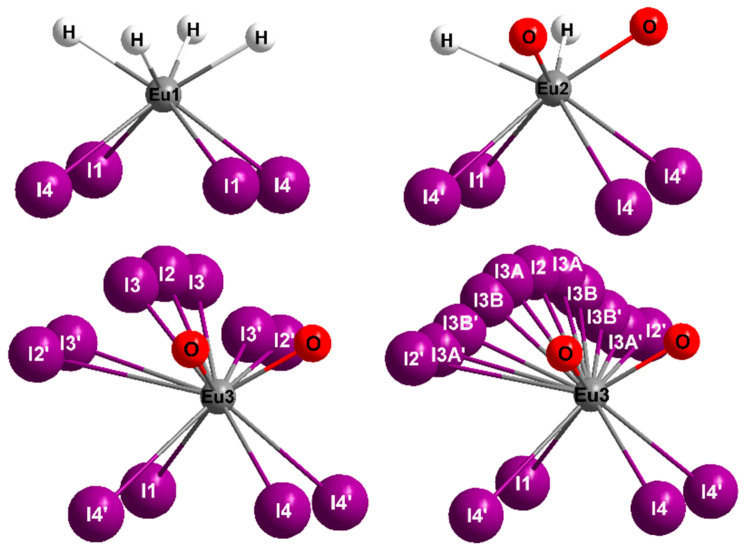
Coordination polyhedra of the Eu^2+^ cations in the crystal structure of Eu_5_H_2_O_2_I_4_, (Eu3)^2+^-centered polyhedra for 100 K (**left**) and 293 K (**right**).

The crystal structure of Eu_5_H_2_O_2_I_4_ contains three crystallographically different Eu^2+^ cations. (Eu1)^2+^ is surrounded by four hydride and four iodide anions, forming a distorted square antiprism with (Eu1)–H distances of 245 pm and (Eu1)–I contacts between 341 and 349 pm (Figure 2, top, and Table 2, all distances mentioned in the text apply to the measurement 100 K). (Eu2)^2+^ shows a square antiprismatic coordination sphere as well, but here, one square of the polyhedron is built up by two *cis*-oriented hydride (*d*((Eu2)−H) = 249 pm) and oxide anions each (Figure 2, top). These bond lengths correspond well with the Eu–H distances of the also tetrahedrally coordinated hydride anions in the hydride halides EuHCl (248 pm) [17] and EuHBr (250 pm) [35], as well as Eu_2_H_3_I (244–250 pm) [18,34].

The same applies to the observed Eu–O bond length of 238 pm being very similar to the corresponding distances in the europium(II) oxide iodides Eu_4_OI_6_ (240 pm) [36] and Eu_2_OI_2_ (237 pm) [37]. The surrounding of (Eu3)^2+^ displays four iodide anions, which are located on fully occupied sites, and two oxide anions at a distance of 233 pm, which is astonishingly short for a Eu^2+^–O^2−^ contact. The coordination sphere is completed by disordered iodide anions in two partially occupied positions. These are located in a distance interval of 324–395 pm for each (Eu3)^2+^ cation (Figure 2, bottom).

**Figure 3:**
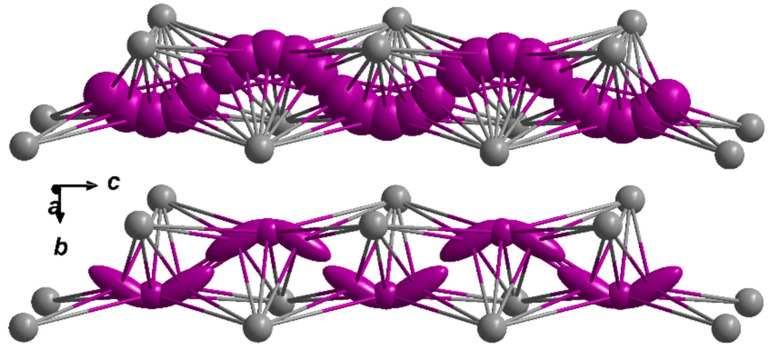
Coordination environment of the disordered I^−^ anions (I2 and I3, violet) by Eu^2+^ cations (grey) in the crystal structure of Eu_5_H_2_O_2_I_4_ at 293 K (**top**) and 100 K (**bottom)**; here, the iodide anions are drawn in an ellipsoid representation at a 95% probability level.

The distances between the disordered iodide anions themselves range between 82 and 343 pm, thus being too short to justify a full occupation of the corresponding sites. When considering the refined site occupation factors (Table 2), a reasonable coordination number of eight for (Eu3)^2+^ is obtained as well. The longer I2⋯I3 contacts with 264 and 343 pm are comparable with the bond length in iodine molecules (*d*(I–I) = 272 pm in solid iodine at 100 K [38]). This would even allow the interpretation of incorporated diatomic iodine (I2) in the compound, which also could explain the observed colour and absorption. The coordination environment of the disordered iodide anions is shown in Figure 3.

While the (I2)^−^ anions appear relatively spherical, the (I3)^−^ anions are strongly elongated parallel to the *bc* plane (see Table 3 for their anisotropic displacement parameters at 100 K), resulting in a banana-shaped displacement ellipsoid. One of these “bananas” corresponds to approximately one iodide anion in total, which is surrounded by six Eu^2+^ cations, forming a strongly distorted trigonal prism. The site occupation factors (Table 2) suggest a higher probability of iodine on the position of the (I3)^−^ anion, which is also supported by the four short (Eu3)^2+^–(I3)^−^ distances of 324 and 342 pm, while the (I2)^−^ anion has only two short contacts to (Eu3)^2+^ cations (328 pm) and the four others are significantly longer (395 pm). The dominating structural feature in the crystal structure of Eu_5_H_2_O_2_I_4_ are the hydride and oxide anion-centred (Eu^2+^)_4_ tetrahedra, [HEu_4_]^7+^ und [OEu_4_]^6+^, which are connected via common edges, forming infinite ribbons parallel to [001].

**Figure 4:**
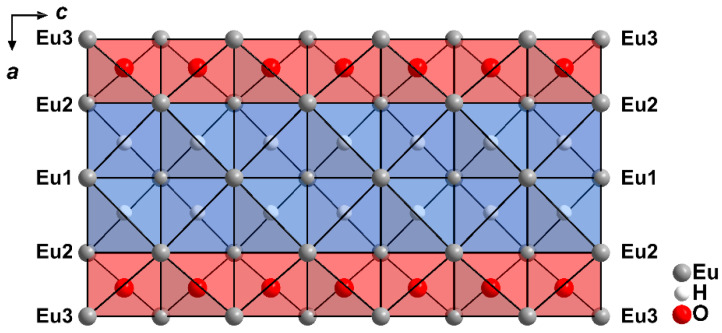
Cationic $\overset{1}{\infty }${[Eu_5_H_2_O_2_]^4+^} ribbons running along [001] in the crystal structure of Eu_5_H_2_O_2_I_4_.

Four trans-edge connected tetrahedra, two hydrogen-centred ones in the inner part of the bands, and two oxygen-centred ones at their outer sides represent the smallest repeating unit parallel to [100] within these $\overset{1}{\infty }${[Eu_5_H_2_O_2_]^4+^} ribbons (Figure 4).

**Figure 5:**
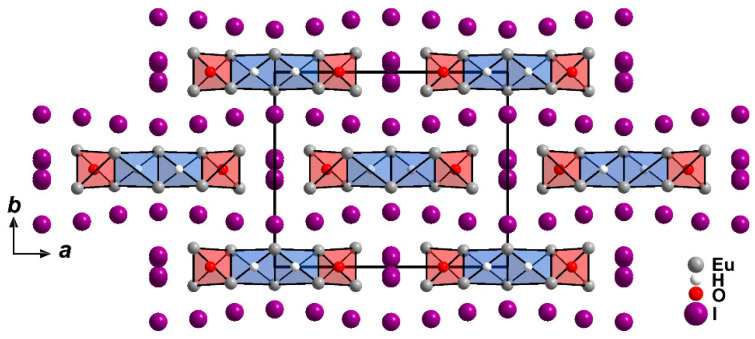
Extended unit-cell content of Eu_5_H_2_O_2_I_4_ at 100 K as viewed along [001].

These positively charged bands are held together and charge-balanced by the ordered iodide anions parallel to [010], and by the disordered ones in the [100] direction. Parallel to the *ab* plane (001) the $\overset{1}{\infty }${[Eu_5_H_2_O_2_]^4+^} ribbons are arranged like bricks in a wall, and the iodide anions serve as mortar between them (Figure 5).

### 2.2. Microprobe Analyses

**Figure 6:**
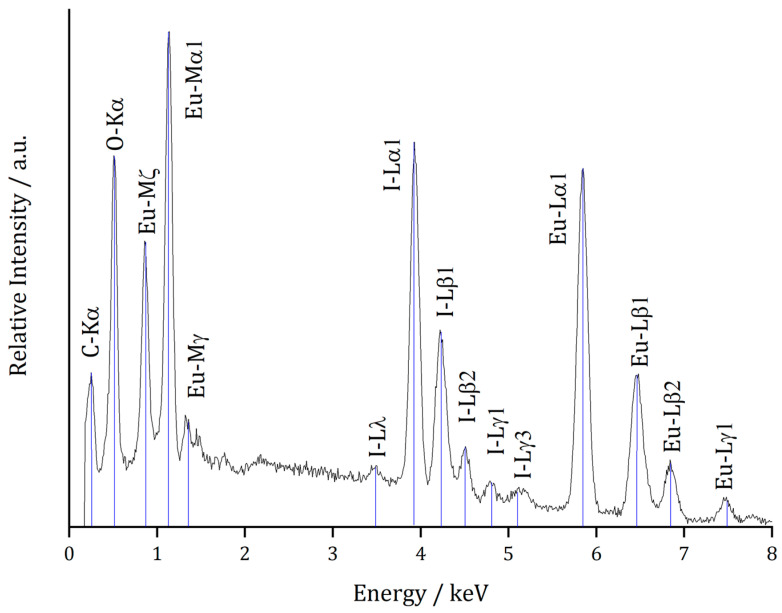
Energy-dispersive X-ray spectrum (*EDXS*) of Eu_5_H_2_O_2_I_4_, with characteristic emission lines of europium and iodine added.

To confirm the described Eu:I ratio of 5:4, a single crystal of Eu_5_H_2_O_2_I_4_ was selected for a wavelength-dispersive X-ray spectroscopic (*WDXS*) measurement. The determined ratio was corrected for the oxidation state of europium, the hydrogen content, which can not be detected by this method, and the resulting amount of oxygen, which is not determined directly. The analysed europium, oxygen, and iodine contents along with the corresponding characteristic emission lines are given in Table 4. Figure 6 shows the (energy-dispersive) *EDX* spectrum for Eu_5_H_2_O_2_I_4_ with the characteristic emission lines added. The observed C-*K*_α_ peak originates from sputtering the sample with carbon to enhance the electrical conductivity for the measurement. The determined Eu:I ratio of approximately 1:1.25 is close to the crystallographically calculated ratio of 5:4 with respect to the potential errors, such as instrumental limitations and sample decomposition due to exposure to air before and after sputtering with carbon.

### 2.3. Luminescence

**Figure 7:**
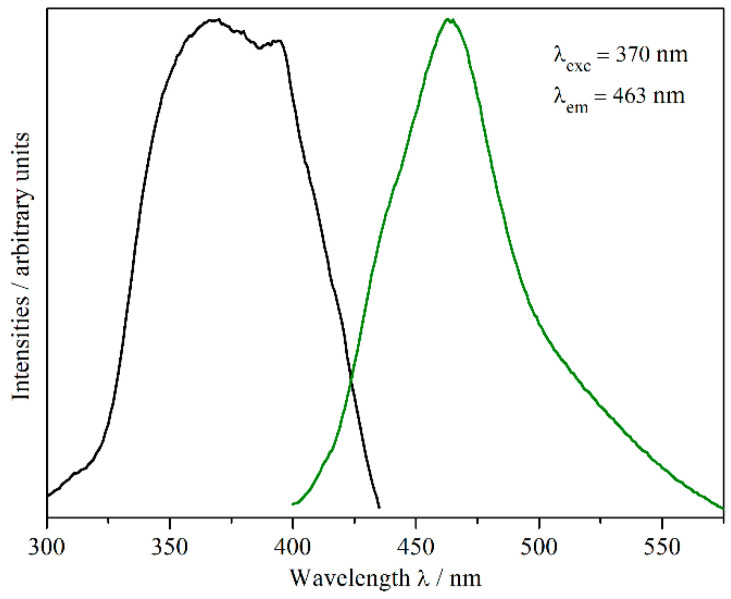
Excitation (black) and emission spectra (green) of single crystals of Eu_5_H_2_O_2_I_4_ (the peak in the excitation spectrum originates from the lamp used for excitation).

The dark red single crystals of Eu_5_H_2_O_2_I_4_ show blue–green luminescence under excitation with UV light (Figure 1). The excitation and emission spectra (Figure 7) exhibit maxima at 370 and 463 nm, respectively, corresponding to a Stokes shift of about 5430 cm^−1^ (=0.67 eV), which is typical of Eu^2+^ coordinated by ligands with a strong nephelauxetic effect.

**Figure 8:**
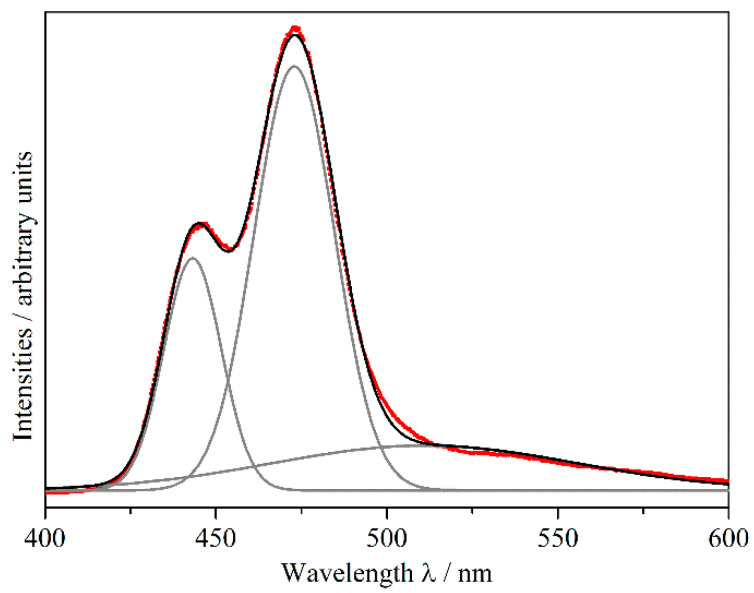
Deconvolution of the measured emission spectrum (red squares) at 30 K, excited with a pulsed laser (λ = 370 nm) on single crystals of Eu_5_H_2_O_2_I_4_ using three Gauß curves (grey), which are summarized in the black curve.

Both excitation and emission are characterized by a broad band, which can be assigned to the [Xe]4*f*^7^–[Xe]4*f*^6^5*d*^1^ transition of the Eu^2+^ cation. The unusual shape of the emission band might be explained by the existence of three crystallographically different Eu^2+^ cations in the crystal structure of Eu_5_H_2_O_2_I_4_, with significantly different coordination surroundings (Section 2.1). Figure 8 shows a deconvolution of the emission curve at 30 K using three Gauß functions.

**Figure 9:**
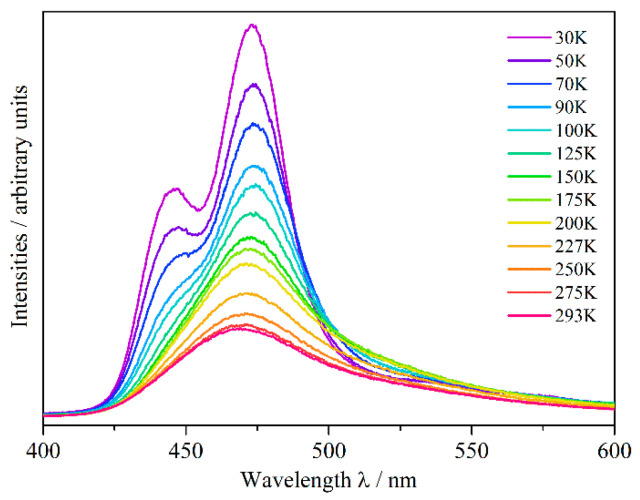
Temperature-dependent emission spectra of single crystals of Eu_5_H_2_O_2_I_4_ excited with a pulsed laser (λ = 370 nm).

The (Eu3)^2+^ cation is only coordinated by weak O^2−^ and I^−^ ligands, which, in addition to the weak nephelauxetic effect of the O^2−^ anions, may lead to an emission at around 443 nm at 30 K. This corresponds to a common emission wavelength of Eu^2+^ in binary halides and oxide halides [39]. With the same coordination number of eight, in the coordination sphere of the (Eu2)^2+^ cation, two iodides are substituted by two hydride ligands as compared to the (Eu3)^2+^-centred coordination sphere. This may cause an emission at a higher wavelength of about 473 nm at 30 K, which is due to the strong nephelauxetic effect (covalency between Eu^2+^ and its ligands) [40,41] of the hydride anion, and a large ligand-field splitting because of its nature as a strong ligand [42]. The emission band decreases only slowly with increasing wavelength, because of a further adjacent maximum resulting from (Eu1)^2+^. This cation, being coordinated by four hydride and four iodide anions and thus surrounded by the most hydride anions of all three Eu^2+^ cations in Eu_5_H_2_O_2_I_4_, should provoke the widest red-shifted emission of the different Eu^2+^ cations in this compound, with an emission maximum at around 511 nm at 30 K. This is comparable with the emission wavelengths of the europium(II) hydride halides EuHCl (510 nm) [17], Eu_2_H_3_Cl (503 nm) [18], and EuHBr (493 nm) [18], with similar coordination spheres and numbers, while EuHI [32] was never again obtained. Hence, its luminescence properties could not be determined. The lifetime of the excited state of Eu^2+^ in Eu_5_H_2_O_2_I_4_ at 30 K is 390 ns (Figure S5, ESIy), and thus it falls into the range of typical europium(II)-doped hydrides [13]. Figure 9 shows the temperature dependence of the photoluminescence emission of Eu_5_H_2_O_2_I_4_ excited by a pulsed laser at 370 nm.

It can be seen that with increasing temperature, the distinct emission bands resulting from the three crystallographically independent sites are not resolved anymore, and appear as one broad band, which is a normal temperature-dependent behaviour of emission bands [43,44,45]. From the decrease in intensities, a quenching temperature (*T*_50%_) of about 110 K can be estimated. This is, however, only a crude estimate, since the emission bands of Eu^2+^ from different crystallographic sites overlap. Further analyses of the three unique emission bands can be found in Figures S2–S4, ESIy. With its emission maximum at 463 nm at room temperature, Eu_5_H_2_O_2_I_4_ shows the shortest Eu^2+^ luminescence emission of all known hydride compounds, which is in contrast to the stronger red-shifted emission in the pure Eu^2+^-doped alkaline-earth metal hydrides *AE*H_2_ (emission maxima: 728–764 nm for *AE* = Ca − Ba [46]) or the hydrogen-rich hydride chlorides *AE*_7_H_12_Cl_2_:Eu^2+^ (emission maxima: 585 nm for *AE* = Sr and 606 nm for *AE* = Ca [19]). This is due to the aforementioned strong nephelauxetic effect of the hydride anion and a large ligand-field splitting of the 5*d* levels of Eu^2+^ in all these compounds. Compared with the short emission wavelength for Eu_5_H_2_O_2_I_4_, the hydride oxide chloride LiEu_2_HOCl_2_ [14] shows a much longer emission wavelength, despite also having a low hydride content. The Eu^2+^ surrounding of LiEu_2_HOCl_2_ is similar to that one of (Eu2)^2+^ in Eu_5_H_2_O_2_I_4_, but with one additional halide cap increasing the coordination number to nine, while the H^−^ anions are coordinated octahedrally by four Eu^2+^ and two Li^+^ cations. A reason for these strongly deviating emission wavelengths might be the different polarisation of the anions by the cations in both compounds, since in LiEu_2_HOCl_2_, there are also monovalent Li^+^ cations next to the divalent Eu^2+^ cations. This could vary the covalent bonding scenario of the europium(II)-ligand bonds and thus influence the red shift of the emission [39]. Another explanation could be the effect of the second coordination sphere on the ligand–field splitting and consequently on the red shift of the Eu^2+^ emission [47].

### 2.4. Magnetism

**Figure 10:**
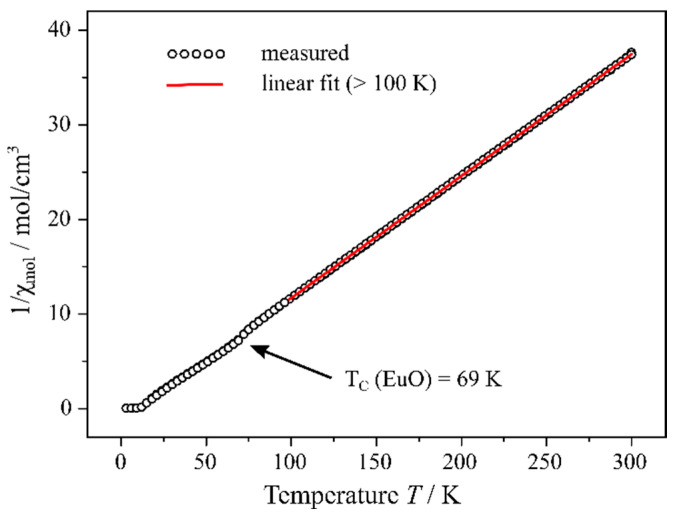
Inverse molar susceptibility of Eu_5_H_2_O_2_I_4_ plotted against temperature.

At temperatures higher than 100 K, Eu_5_H_2_O_2_I_4_ reveals a Curie behaviour, showing the typical linear dependence between the inverse magnetic susceptibility and temperature (Figure 10).

**Figure 11:**
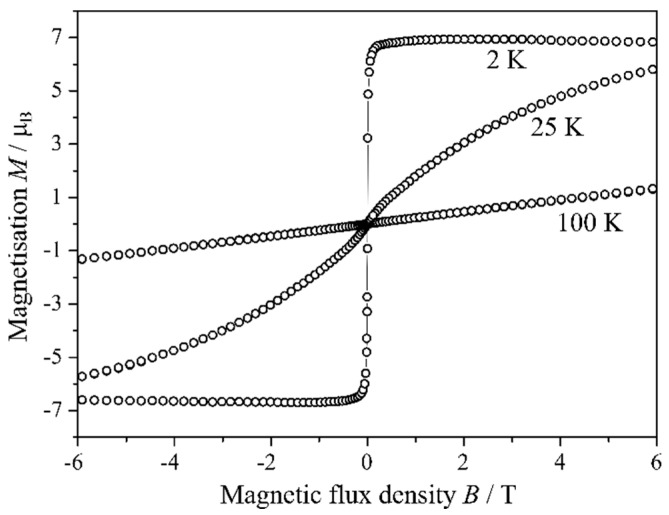
Hysteresis loops of Eu_5_H_2_O_2_I_4_ at 2, 25, and 100 K.

A linear fit of the obtained values results in an experimental magnetic moment of 7.88 (1) µ_B_ for one europium cation, which is very close to the theoretical value of 7.94 µ_B_ for an isolated Eu^2+^ cation with 4*f*^7^ configuration, while Eu^3+^ would show a completely different behaviour [48]. At lower temperatures, at first, an irregularity at about 70 K occurs, which is probably due to small impurities of europium(II) oxide, showing its ferromagnetic transition (*T*_C_(EuO) = 69 K [49]). At temperatures below 12 K, the magnetic susceptibility of Eu_5_H_2_O_2_I_4_ rises steeply up to a magnetic saturation at 8 K. For the inverse magnetic susceptibility in Figure 10, this is reflected by the fact that for these values, a minimum is achieved. The latter observation leads to the assumption that Eu_5_H_2_O_2_I_4_ has a ferromagnetic transition at about 10 K, and the results of the hysteresis measurements (Figure 11) confirm this assumption. While at temperatures of 100 and 25 K, a typical pa-ramagnetic behaviour of Eu_5_H_2_O_2_I_4_ is observed, at 2 K, the characteristics of a weak ferromagnetic material appear. The presented magnetic properties of Eu_5_H_2_O_2_I_4_ support the existence of only Eu^2+^ in the compound; however, small amounts of incorporated Eu^3+^ can not be completely excluded with this method.

## 3. Experimental Procedure

### 3.1. Motivation

After the successful synthesis and characterisation of matlockite-type EuHCl [17] and EuHBr [18,35] several years ago, our target was to prepare doubtful EuHI [32] unequivocally from 1:1-molar mixtures of EuH_2_ and EuI_2_. Due to the air- and moisture-sensitivity of all starting materials and products, they were carefully handled in an argon-filled glove box (MBraun).

### 3.2. Synthesis

Up to millimetre-long, dark red single crystals of the europium(II) hydride oxide iodide Eu_5_H_2_O_2_I_4_ (Figure 1) were obtained through the reaction of equimolar amounts of oxygen-contaminated europium(II) hydride (EuH_2_: self-made by hydrogenation of europium pieces at 500 °C; Eu: ChemPur, 99.9%, H_2_: Linde, 99.9%) and europium(II) iodide (EuI_2_: Sigma Aldrich, 99.9%) in a sodium-iodide flux (NaI: Merck, ultrapure) while attempting to synthesize single crystals of the europium(II) hydride iodide EuHI described in the literature [32]. Niobium capsules self-made from niobium tubes (Sigma-Aldrich) and arc-welded under a helium atmosphere served as the container material. In order to prevent their oxidation, they were enclosed in evacuated fused silica ampoules. The reaction mixtures were heated to 900 °C within 12 h, kept at this temperature for 24 h, and cooled down to room temperature within 48 h. The mostly inhomogeneous initial product mixtures (Figure 1) consisted of dark red Eu_5_H_2_O_2_I_4_ (main component), alongside Eu_2_OI_2_ (orange) and Eu_4_OI_6_ (yellow), as well as fluxing NaI (white). Attempts to prepare phase-pure Eu_5_H_2_O_2_I_4_ from EuH_2_, EuO (self-made from europium metal and Eu_2_O_3_: ChemPur, 99.9%) and EuI_2_ never succeeded, so the best results with yields up to 75% Eu_5_H_2_O_2_I_4_ were always gained by NaI-flux-assisted reactions of self-made oxygen-contaminated europium dihydride (EuH_2−*x*_O_0.5*x*_ with various x) with equimolar amounts of commercially available europium diiodide (EuI_2_) in a slight excess, as compared to the stoichiometrically necessary portion.

### 3.3. X-ray Diffraction

By using a light microscope (Leica) in the inert argon atmosphere of a glove box (MBraun), suitable single crystals of Eu_5_H_2_O_2_I_4_ for X-ray diffraction experiments could be selected and put into Lindemann glass capillaries (Hilgenberg). After a first measurement at room temperature (293 K), the diffraction intensities were collected again at 100 K, because the crystal structure showed disordered iodide anions at room temperature, but their disorder could be reduced at lower temperature (Section 3.1). A κ-CCD diffractometer (Bruker-Nonius) with graphite-monochromatised Mo-*K*α radiation (λ = 71.07 pm) was used for the collection of both intensity data sets. After applying an empirical absorption correction with the program *SCALEPACK* [50], the structure solution and refinement (full-matrix least-squares against *F*^2^) was carried out with the program package [51,52]. The structure was solved by direct methods with anisotropic displacement factors for all non-hydrogen atoms. The position of the hydrogen atom could be taken from the list of the remaining residual electron density maxima and refined by constraining the isotropic displacement factor to the parameter of the oxygen atom. The corresponding crystallographic results are summarized in Table 1, Table 2 and Table 3.

### 3.4. Microprobe

Wavelength-dispersive X-ray spectroscopy (*WDXS*) and energy-dispersive X-ray spectroscopy (*EDXS*) measurements were carried out using an electron beam microprobe device SX100 from Cameca (Gennevilliers, France). As a reference for europium, monazite-type Eu[PO_4_] (LLIF crystal) was used, while iodine was referenced using a crystal of potassium iodide KI (LPET crystal).

### 3.5. Luminescence

For the photoluminescence measurements, single crystals of Eu_5_H_2_O_2_I_4_ were selected under a light microscope embedded in the glove box. Due to their air- and moisture-sensitivity, the single crystals were enclosed into silica ampoules (diameter: 5 mm, length: 35 mm). Excitation and emission spectra were measured with a Horiba FluoroMax-4 fluorescence spectrometer equipped with a xenon discharge lamp at room temperature. The temperature-dependent luminescence was measured with a tuneable optical parametric oscillator pumped by a neodymium-YAG laser (Ekspla NT342B-SH with 6 ns pulse lengths) together with a Jobin-Yvon HR250 monochromator (600 grooves/mm) and a PI-MAX ICCD camera (Princeton Instruments) for detection [50]. Accumulations were collected per measurement to increase the signal-to-noise ratio. The samples were placed into a Janis closed-cycle helium cryostat with a Lakeshore temperature controller, and were fixed to the cold finger using high-purity silver paint and copper tape. Decay measurements were recorded with the same set-up. Data were recorded 50 ns after the laser pulse with up to 3 ms delays, with an integration window of 25 ns.

### 3.6. Magnetism

For measurements of the magnetic properties, polycrystalline samples of Eu_5_H_2_O_2_I_4_ (as a mixture with the fluxing agent NaI) were placed into gelatine capsules and attached to the sample holder of a Vibrating Sample Magnetometer (*VSM*) for measuring the magnetizations *M*(*T*) and *M*(*H*) in a Magnetic Property Measurement System (MPMS3, Quantum Design, USA). For *M*(*T*) measurements, the samples were examined within a temperature range from 2 to 300 K in a homogeneous magnetic field of 500 Oe; for *M*(*H*), data hysteresis loops (−7 T ≤ *H* ≥ +7 T) at 100, 25 and 2 K have been recorded.

## 4. Conclusions

So far, the emission maxima of the Eu^2+^-centred luminescence in hydride materials range in the red region of the electromagnetic spectrum for pure dihydrides. Since EuH_2_ does not luminesce as bulk, the Eu^2+^-doped alkaline-earth metal dihydrides *AE*H_2_ (*AE* = Ca − Ba) with their cotunnite-type structures (*C.N*.(*M*^2+^) = 9) need to serve as landmarks. Upon switching to the Eu^2+^-doped hydrogen-rich hydride chlorides *AE*_7_H_12_Cl_2_ (*AE* = Ca and Sr, *C.N*.(*M*^2+^) = 9), a blue-shift to orange occurs, which even turns to green for the bulk matlockite-type hydride chlorides EuH*X* (*X* = Cl and Br, *C.N*.(*M*^2+^) = 9) and Eu_2_H_3_Cl (*C.N*.(*M*^2+^) = 10). In hitherto unsuccessful attempts to obtain the iodide analogue EuHI, oxygen contamination led to the serendipitous formation of the europium(II) hydride oxide iodide hydride Eu_5_H_2_O_2_I_4_ (*C.N*.(*M*^2+^) = 8), which shows a blue-green bulk luminescence at 463 nm (λ_exc_ = 370 nm). This represents the Eu^2+^ phosphor with the shortest emission wavelength among all europium(II)-hydride derivatives. Using photoluminescence spectroscopy, the influence of the different coordination environments around the crystallographically distinguishable Eu^2+^ cations becomes evident, when the shape of the emission spectrum is considered. Furthermore, temperature-dependent measurements also showed an additional emission peak at around 443 nm, which seems to arise from the oxygen-rich site (Eu3)^2+^, as oxide anions only show a weak nephelauxetic effect. As the 4*f*^7^-configuration of the Eu^2+^ cations may also introduce interesting magnetic effects, the magnetic susceptibility was determined in the range of 2 to 300 K. While the title compound Eu_5_H_2_O_2_I_4_ shows a paramagnetic behaviour above 10 K, a ferromagnetic transition was observed towards lower temperatures. Additional magnetic hysteresis measurements confirmed a weak ferromagnetic ordering when measured at 2 K.

## Figures and Tables

**Table 1:** Crystallographic data and their determination for the crystal structure of Eu_5_H_2_O_2_I_4_ at 100 and 293 K.

Chemical formula	Eu_5_H_2_O_2_I_4_	
Molar mass, M/g·mol^−1^	1301.41	
Crystal system	orthorhombic	
Space group	*Cmcm* (no. 63)	
Measuring temperature, *T*/K	100 (2)	293 (2)
*a*/pm	1636.97 (9)	1642.51 (9)
*b*/pm	1369.54 (8)	1374.23 (8)
*c*/pm	604.36 (4)	606.58 (4)
$\mathrm{Molar}\;\mathrm{volume},\;V_{m}/{\mathrm{cm}}^{3}\cdot$mol^−1^	203.98 (2)	206.15 (2)
Number of formula units, *Z*	4	
Number of measured reflections	2931	1618
Number of independent reflections	1645	921
*wR*_2_	0.092	0.106
*R*_1_	0.042	0.041
Goodness of Fit	1.090	1.068
CSD number	434116	434115

**Table 2:** Fractional atomic coordinates and site occupation factors (*s. o. f.*) for Eu_5_H_2_O_2_I_4_, top: 100 K, bottom: 293 K.

Atom	Site	*s. o. f.*	*x*/*a*	*y*/*b*	*z*/*c*
Eu1	4*c*	1	0	0.90892 (4)	^1^/_4_
Eu2	8*g*	1	0.18814 (2)	0.08380 (3)	^1^/_4_
Eu3	8*g*	1	0.15203 (2)	0.40322 (3)	^1^/_4_
H	8*e*	1	0.090 (7)	0	0
O	8*e*	1	0.2762 (3)	0	0
I1	4*c*	1	0	0.21783 (6)	^1^/_4_
I2	4*c*	0.232 (15)	0	0.5589 (4)	^1^/_4_
I3	8*f*	0.377 (9)	0	0.5436 (4)	0.1192 (15)
I4	8*g*	1	0.32809 (3)	0.26778 (4)	^1^/_4_
Eu1	4*c*	1	0	0.90947 (6)	^1^/_4_
Eu2	8*g*	1	0.18819 (4)	0.08329 (4)	^1^/_4_
Eu3	8*g*	1	0.15234 (4)	0.40378 (4)	^1^/_4_
H	8*e*	1	0.089 (9)	0	0
O	8*e*	1	0.2766 (5)	0	0
I1	4*c*	1	0	0.21795 (9)	^1^/_4_
I2	4*c*	0.284 (11)	0	0.5601 (5)	^1^/_4_
I3^A^	8*f*	0.212 (8)	0	0.5503 (4)	0.1422 (11)
I3^B^	8*f*	0.150 (7)	0	0.5234 (7)	0.0577 (16)
I4	8*g*	1	0.32802 (5)	0.26771 (7)	^1^/_4_

^A^ and ^B^: I3^A^ and I3^B^ represent split positions in the room-temperature measurement (**bottom**), which coincide into I3 in the low-temperature case (**top**).

**Table 3:** Anisotropic and equivalent isotropic displacement parameters (*U_ij_* and *U_eq_* ^(a)^ in pm^2^) for Eu_5_H_2_O_2_I_4_, top: 100 K, bottom: 293 K.

Atom	*U*_11_	*U*_22_	*U*_33_	*U*_23_	*U*_13_	*U*_12_	*U*_eq_
Eu1	62 (2)	25 (2)	133 (3)	0	0	0	73 (1)
Eu2	43 (2)	8 (2)	98 (2)	0	0	7 (1)	50 (1)
Eu3	31 (2)	31 (2)	14 (1)	0	0	−11 (1)	67 (1)
H	–	–	–	–	–	–	191 ^(b)^
O	67 (22)	38 (22)	125 (26)	−7 (20)	0	76 (10)	76 (10)
I1	45 (3)	100 (3)	99 (3)	0	0	0	81 (2)
I2	130 (18)	176 (20)	196 (52)	0	0	0	167 (21)
I3	142 (9)	391 (17)	888 (51)	417 (27)	0	0	474 (21)
I4	49 (2)	58 (2)	120 (2)	0	0	−29 (2)	76 (1)
Eu1	131 (5)	123 (4)	262 (5)	0	0	0	172 (3)
Eu2	113 (4)	87 (4)	224 (4)	0	0	7 (2)	141 (2)
Eu3	117 (4)	133 (4)	294 (4)	0	0	−32 (2)	181 (3)
H	–	–	–	–	–	–	516 ^(b)^
O	210 (40)	160 (40)	260 (40)	−60 (30)	0	0	206 (18)
I1	167 (6)	218 (6)	262 (6)	0	0	0	216 (3)
I2	–	–	–	–	–	–	394 (25) ^(c)^
I3^A^	–	–	–	–	–	–	267 (20) ^(c)^
I3^B^	–	–	–	–	–	–	402 (29) ^(c)^
I4	160 (4)	186 (5)	328 (5)	0	0	−53 (3)	225 (3)

^(a)^ Ueq = 1/3 [U11 + U22 + U33], ^(b)^ the isotropic displacement parameter of the hydrogen atom was constrained to the parameter of the oxygen atom (factor: 2.5), ^(c)^ Uiso values; ^A^ and ^B^: for I3^A^ and I3^B^ see footnote in Table 2.

**Table 4:** Quantitative electron beam microprobe analysis for Eu_5_H_2_O_2_I_4_.

Ion	Emission Line (Standard)	Content/wt.-%	Normalized Content/at.-%
Eu^2+^	*L*_α_ (Eu[PO_4_])	39.8 (2)	41.7 (6)
I^−^	*L*_α_ (KI)	26.3 (2)	33.1 (4)
O^2−^	–	5.9 (4)	25.2 (2)

## Data Availability

Not applicable.

## Supplementary Materials

The following supporting information can be downloaded at: https://www.mdpi.com/article/10.3390/ijms241914969/s1.
