# Supplementary material for: Synthesis and Crystal Structure of the Europium(II) Hydride Oxide Iodide Eu5H2O2I4 Showing Blue-Green Luminescence
Source: Int J Mol Sci. 2023 Oct 7;24(19):14969. doi: 10.3390/ijms241914969 (PMC10573458; doi:10.3390/ijms241914969)
Supplement: Supplementary file 1 [file ijms-24-14969-s001.zip › ijms-2592018-supplementary.pdf]

Supporting Information

Synthesis and crystal structure of the europium(II) hydride oxide iodide $Eu_5H_2O_2I_4$ showing blue-green luminescence

Daniel Rudolph[1], Thomas Wylezich[2], Philip Netzsch[3], Björn Blaschkowski[1], Henning A. Höppe[3], Philippe Goldner[4], Nathalie Kunkel[5], Jean-Louis Hoslauer[1] and Thomas Schleid[1,*]

[1] *Institut für Anorganische Chemie, Universität Stuttgart, Pfaffenwaldring 55, 70569 Stuttgart, Germany; blaschkowski@iac.uni-stuttgart.de (B.B.)*

[2] *Institut für Anorganische Chemie, Technische Universität München, Lichtenbergstrasse 4, 85747 Garching, Germany; thomas.wylezich@tum.de*

[3] *Institut für Physik, Universität Augsburg, Universitätsstraße 1, 86159 Augsburg, Germany*

[4] *Chimie ParisTech, PSL University, CNRS, Institut de Recherche de Chimie Paris, 75005 Paris, France*

[5] *Institut für Anorganische Chemie, Georg-August-Universität Göttingen, Tammannstrasse 4, 37077 Göttingen, Germany*

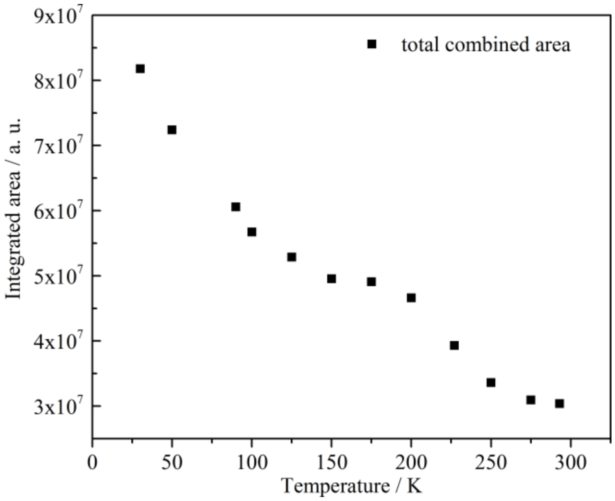

Figure S1. Temperature-dependent intensity of the emission (peak area) of $Eu_5H_2O_2I_4$. The quenching temperature is estimated to be around 110 K.

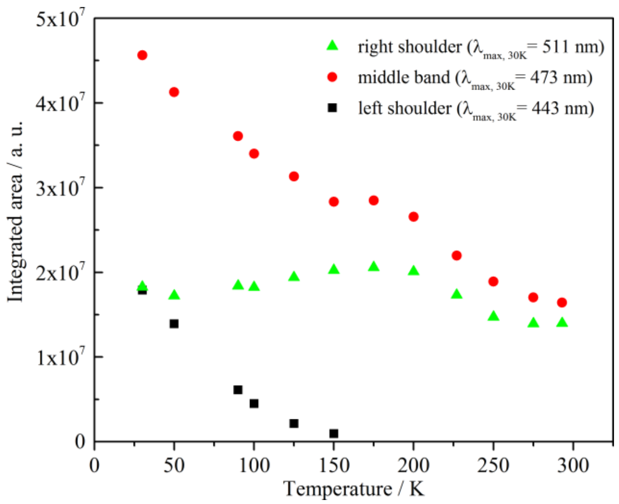

Figure S2. Integrated area of the three emission bands of $Eu_5H_2O_2I_4$ resulting after deconvolution.

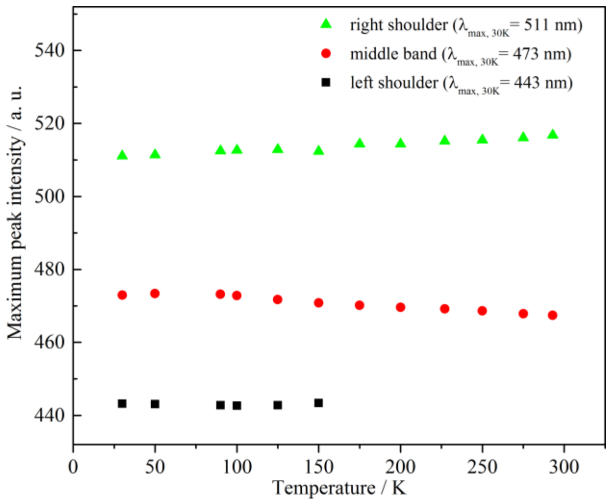

Figure S3. Change of the three individual emission positions in $Eu_5H_2O_2I_4$ with temperature.

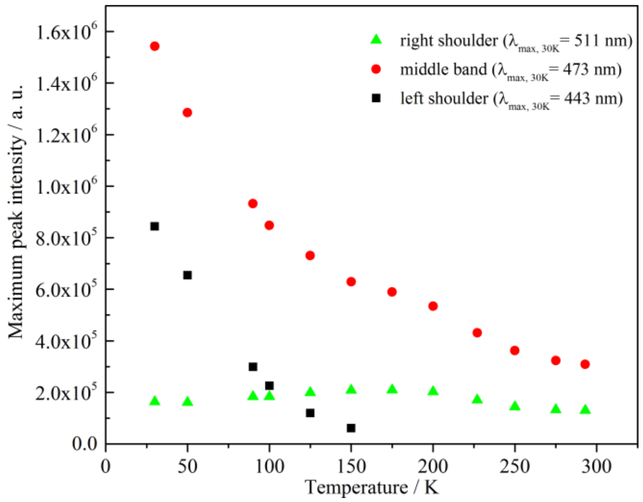

Figure S4. Maximum peak intensity of the three emission bands in $Eu_5H_2O_2I_4$ after deconvolution.

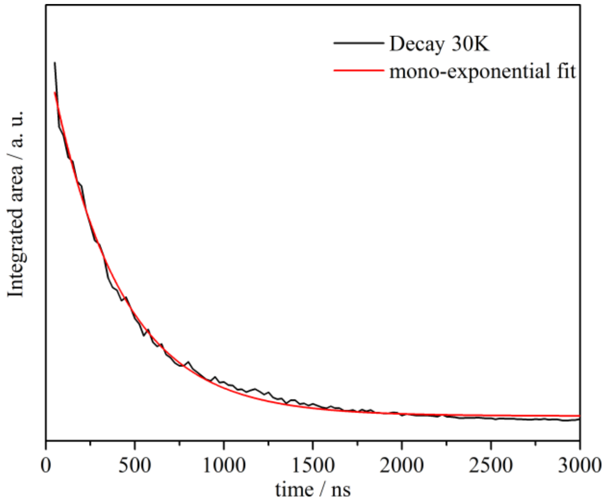

Figure S5. Decay curve of $Eu_5H_2O_2I_4$ at 30 K. Fitting of the parameters using a mono-exponential decay results in a lifetime of the excited state of 388±5 ns.

```
_audit_creation_method           SHELXL-97
_chemical_name_systematic
;
 ?
;
_chemical_name_common            ?
_chemical_melting_point          ?
_chemical_formula_moiety         ?
_chemical_formula_sum
 'Eu5 H2 I4 O2'
_chemical_formula_weight         1301.42

loop_
 _atom_type_symbol
 _atom_type_description
 _atom_type_scat_dispersion_real
 _atom_type_scat_dispersion_imag
 _atom_type_scat_source
 'Eu'  'Eu'  -0.1578   3.6682
 'International Tables Vol C Tables 4.2.6.8 and 6.1.1.4'
 'H'  'H'   0.0000   0.0000
 'International Tables Vol C Tables 4.2.6.8 and 6.1.1.4'
 'O'  'O'   0.0106   0.0060
 'International Tables Vol C Tables 4.2.6.8 and 6.1.1.4'
 'I'  'I'  -0.4742   1.8119
 'International Tables Vol C Tables 4.2.6.8 and 6.1.1.4'

_symmetry_cell_setting           orthorhombic
_symmetry_space_group_name_H-M    Cmcm

loop_
 _symmetry_equiv_pos_as_xyz
 'x, y, z'
 '-x, -y, z+1/2'
 'x, -y, -z'
 '-x, y, -z+1/2'
 'x+1/2, y+1/2, z'
 '-x+1/2, -y+1/2, z+1/2'
 'x+1/2, -y+1/2, -z'
 '-x+1/2, y+1/2, -z+1/2'
 '-x, -y, -z'
 'x, y, -z-1/2'
 '-x, y, z'
 'x, -y, z-1/2'
 '-x+1/2, -y+1/2, -z'
 'x+1/2, y+1/2, -z-1/2'
 '-x+1/2, y+1/2, z'
```

'x+1/2, -y+1/2, z-1/2'

_cell_length_a 16.4251(9)
_cell_length_b 13.7423(8)
_cell_length_c 6.0658(4)
_cell_angle_alpha 90.00
_cell_angle_beta 90.00
_cell_angle_gamma 90.00
_cell_volume 1369.16(14)
_cell_formula_units_Z 4
_cell_measurement_temperature 293(2)
_cell_measurement_reflns_used 955
_cell_measurement_theta_min 0.41
_cell_measurement_theta_max 27.88

_exptl_crystal_description 'lath shaped'
_exptl_crystal_colour 'red'
_exptl_crystal_size_max 0.160
_exptl_crystal_size_mid 0.081
_exptl_crystal_size_min 0.043
_exptl_crystal_density_meas ?
_exptl_crystal_density_diffrn 6.313
_exptl_crystal_density_method 'not measured'
_exptl_crystal_F_000 2180
_exptl_absorpt_coefficient_mu 31.548
_exptl_absorpt_correction_type empirical
_exptl_absorpt_correction_T_min 0.005
_exptl_absorpt_correction_T_max 0.232
_exptl_absorpt_process_details 'SCALEPACK (W. Minor & Z. Otwinowski)'

_exptl_special_details
;
 ?
;

_diffrn_ambient_temperature 293(2)
_diffrn_radiation_wavelength 0.71069
_diffrn_radiation_type MoK\a
_diffrn_radiation_source 'fine-focus sealed tube'
_diffrn_radiation_monochromator graphite
_diffrn_measurement_device_type 'Kappa CCD (Nonius)'
_diffrn_measurement_method 'four circle, CCD'
_diffrn_detector_area_resol_mean ?
_diffrn_standards_number ?
_diffrn_standards_interval_count ?
_diffrn_standards_interval_time ?
_diffrn_standards_decay_% ?

_diffrn_reflns_number 1618
_diffrn_reflns_av_R_equivalents 0.0564
_diffrn_reflns_av_sigmaI/netI 0.0229
_diffrn_reflns_limit_h_min -21
_diffrn_reflns_limit_h_max 21
_diffrn_reflns_limit_k_min -18
_diffrn_reflns_limit_k_max 18
_diffrn_reflns_limit_l_min -7
_diffrn_reflns_limit_l_max 7
_diffrn_reflns_theta_min 1.93
_diffrn_reflns_theta_max 27.88
_reflns_number_total 921
_reflns_number_gt 863
_reflns_threshold_expression >2sigma(I)

_computing_data_collection 'Nonius Collect-Software'
_computing_cell_refinement 'Denzo (W. Minor & Z. Otwinowski)'
_computing_data_reduction 'Denzo (W. Minor & Z. Otwinowski)'
_computing_structure_solution 'SHELXS-97 (Sheldrick, 1990)'
_computing_structure_refinement 'SHELXL-97 (Sheldrick, 1997)'
_computing_molecular_graphics ?
_computing_publication_material ?

_refine_special_details
;
Refinement of F^2^ against ALL reflections. The weighted R-factor wR and
goodness of fit S are based on F^2^, conventional R-factors R are based
on F, with F set to zero for negative F^2^. The threshold expression of
F^2^ > 2sigma(F^2^) is used only for calculating R-factors(gt) etc. and is
not relevant to the choice of reflections for refinement. R-factors based
on F^2^ are statistically about twice as large as those based on F, and R-
factors based on ALL data will be even larger.
;

_refine_ls_structure_factor_coef Fsqd
_refine_ls_matrix_type full
_refine_ls_weighting_scheme calc
_refine_ls_weighting_details
'calc w=1/[\s^2^(Fo^2^)+(0.0717P)^2^+31.1628P] where P=(Fo^2^+2Fc^2^)/3'
_atom_sites_solution_primary direct
_atom_sites_solution_secondary difmap
_refine_ls_extinction_method SHELXL
_refine_ls_extinction_coef 0.00159(12)
_refine_ls_extinction_expression
'Fc^*^=kFc[1+0.001xFc^2^\l^3^/sin(2\q)]^-1/4^'
_refine_ls_number_reflns 921
_refine_ls_number_parameters 45

_refine_ls_number_restraints 0
_refine_ls_R_factor_all 0.0412
_refine_ls_R_factor_gt 0.0390
_refine_ls_wR_factor_ref 0.1057
_refine_ls_wR_factor_gt 0.1037
_refine_ls_goodness_of_fit_ref 1.068
_refine_ls_restrained_S_all 1.068
_refine_ls_shift/su_max 0.000
_refine_ls_shift/su_mean 0.000

loop_
_atom_site_label
_atom_site_type_symbol
_atom_site_fract_x
_atom_site_fract_y
_atom_site_fract_z
_atom_site_U_iso_or_equiv
_atom_site_adp_type
_atom_site_occupancy
_atom_site_symmetry_multiplicity
_atom_site_calc_flag
_atom_site_refinement_flags
_atom_site_disorder_assembly
_atom_site_disorder_group
Eu1 Eu 0.0000 0.90947(6) 0.2500 0.0172(3) Uani 1 4 d S . .
Eu2 Eu 0.18819(4) 0.08329(4) 0.2500 0.0141(2) Uani 1 2 d S . .
Eu3 Eu 0.15234(4) 0.40378(4) 0.2500 0.0181(3) Uani 1 2 d S . .
O O 0.2766(5) 0.0000 0.0000 0.0206(18) Uani 1 2 d S . .
H H 0.089(9) 0.0000 0.0000 0.052 Uiso 1 2 d S . .
I1 I 0.0000 0.21795(9) 0.2500 0.0216(3) Uani 1 4 d S . .
I2 I 0.0000 0.5601(5) 0.2500 0.039(3) Uiso 0.284(11) 4 d SP . .
I3A I 0.0000 0.5503(4) 0.1422(11) 0.027(2) Uiso 0.212(8) 2 d SP . .
I3B I 0.0000 0.5234(7) 0.0577(16) 0.040(3) Uiso 0.150(7) 2 d SP . .
I4 I 0.32802(5) 0.26771(7) 0.2500 0.0225(3) Uani 1 2 d S . .

loop_
_atom_site_aniso_label
_atom_site_aniso_U_11
_atom_site_aniso_U_22
_atom_site_aniso_U_33
_atom_site_aniso_U_23
_atom_site_aniso_U_13
_atom_site_aniso_U_12
Eu1 0.0131(5) 0.0123(4) 0.0262(5) 0.000 0.000 0.000
Eu2 0.0113(4) 0.0087(4) 0.0224(4) 0.000 0.000 0.00074(18)
Eu3 0.0117(4) 0.0133(4) 0.0294(4) 0.000 0.000 -0.0032(2)
O 0.021(4) 0.016(4) 0.026(4) -0.006(3) 0.000 0.000

I1 0.0167(6) 0.0218(6) 0.0264(6) 0.000 0.000 0.000
I4 0.0160(4) 0.0186(5) 0.0328(5) 0.000 0.000 -0.0053(3)

_geom_special_details
;
All esds (except the esd in the dihedral angle between two l.s. planes)
are estimated using the full covariance matrix. The cell esds are taken
into account individually in the estimation of esds in distances, angles
and torsion angles; correlations between esds in cell parameters are only
used when they are defined by crystal symmetry. An approximate (isotropic)
treatment of cell esds is used for estimating esds involving l.s. planes.
;

loop_
_geom_bond_atom_site_label_1
_geom_bond_atom_site_label_2
_geom_bond_distance
_geom_bond_site_symmetry_2
_geom_bond_publ_flag
Eu1 I4 3.4314(10) 5_455 ?
Eu1 I4 3.4314(10) 15 ?
Eu1 I1 3.5021(8) 9_565 ?
Eu1 I1 3.5021(8) 9_566 ?
Eu1 Eu2 3.9065(9) 1_565 ?
Eu1 Eu2 3.9065(9) 11_565 ?
Eu1 Eu1 3.9229(11) 9_576 ?
Eu1 Eu1 3.9229(11) 9_575 ?
Eu1 Eu2 4.3317(5) 3_566 ?
Eu1 Eu2 4.3317(5) 9_565 ?
Eu1 Eu2 4.3317(5) 9_566 ?
Eu1 Eu2 4.3317(5) 3_565 ?
Eu2 O 2.391(5) . ?
Eu2 O 2.391(5) 10_556 ?
Eu2 I4 3.4202(9) . ?
Eu2 Eu3 3.5981(9) 15_545 ?
Eu2 I1 3.6027(9) . ?
Eu2 I4 3.6690(7) 13_556 ?
Eu2 I4 3.6690(7) 13 ?
Eu2 Eu2 3.7999(7) 3 ?
Eu2 Eu2 3.7999(7) 3_556 ?
Eu2 Eu1 3.9065(9) 1_545 ?
Eu2 Eu3 4.0114(7) 13_556 ?
Eu2 Eu3 4.0114(7) 13 ?
Eu2 H 2.50(10) . ?
Eu3 O 2.326(4) 6 ?
Eu3 O 2.326(4) 13 ?
Eu3 I3B 3.213(4) 10_556 ?

Eu3 I3B 3.213(4) . ?
Eu3 I3A 3.278(3) . ?
Eu3 I3A 3.278(3) 10_556 ?
Eu3 I3B 3.278(5) 9_565 ?
Eu3 I3B 3.278(5) 2_565 ?
Eu3 I2 3.298(4) . ?
Eu3 I4 3.4384(11) . ?
Eu3 I3A 3.510(4) 9_565 ?
Eu3 I3A 3.510(4) 2_565 ?
O Eu3 2.326(4) 15_545 ?
O Eu3 2.326(4) 13 ?
O Eu2 2.391(5) 3 ?
I1 Eu1 3.5021(8) 9_565 ?
I1 Eu1 3.5021(8) 9_566 ?
I1 Eu3 3.5752(10) 11 ?
I1 Eu2 3.6027(9) 11 ?
I2 I3A 0.667(7) 10_556 ?
I2 I3A 0.667(7) . ?
I2 I3B 1.271(12) . ?
I2 I3B 1.271(12) 10_556 ?
I2 I3B 2.191(12) 9_565 ?
I2 I3B 2.191(12) 2_565 ?
I2 I3A 2.822(8) 9_565 ?
I2 I3A 2.822(8) 2_565 ?
I2 Eu3 3.298(4) 11 ?
I3A I3B 0.632(9) . ?
I3A I3A 1.308(13) 10_556 ?
I3A I3B 1.580(17) 9_565 ?
I3A I3B 1.858(15) 10_556 ?
I3A I3A 2.211(14) 9_565 ?
I3A I3B 2.716(10) 2_565 ?
I3A I2 2.822(8) 9_565 ?
I3A Eu3 3.278(3) 11 ?
I3A Eu3 3.510(4) 9_565 ?
I3A Eu3 3.510(4) 3_565 ?
I3B I3B 0.95(2) 9_565 ?
I3B I3A 1.580(17) 9_565 ?
I3B I3A 1.858(15) 10_556 ?
I3B I2 2.191(12) 9_565 ?
I3B I3B 2.33(2) 10_556 ?
I3B I3A 2.716(10) 2_564 ?
I3B I3B 3.100(4) 2_564 ?
I3B I3B 3.100(4) 2_565 ?
I3B Eu3 3.213(4) 11 ?
I4 Eu1 3.4314(10) 5_545 ?
I4 Eu2 3.6690(7) 13_556 ?
I4 Eu2 3.6690(7) 13 ?

```
loop_
 _geom_angle_atom_site_label_1
 _geom_angle_atom_site_label_2
 _geom_angle_atom_site_label_3
 _geom_angle
 _geom_angle_site_symmetry_1
 _geom_angle_site_symmetry_3
 _geom_angle_publ_flag
I4 Eu1 I4 110.82(4) 5_455 15 ?
I4 Eu1 I1 73.510(19) 5_455 9_565 ?
I4 Eu1 I1 73.510(19) 15 9_565 ?
I4 Eu1 I1 73.510(19) 5_455 9_566 ?
I4 Eu1 I1 73.510(19) 15 9_566 ?
I1 Eu1 I1 120.00(5) 9_565 9_566 ?
I4 Eu1 Eu2 176.90(3) 5_455 1_565 ?
I4 Eu1 Eu2 72.286(19) 15 1_565 ?
I1 Eu1 Eu2 107.802(11) 9_565 1_565 ?
I1 Eu1 Eu2 107.802(11) 9_566 1_565 ?
I4 Eu1 Eu2 72.286(19) 5_455 11_565 ?
I4 Eu1 Eu2 176.90(3) 15 11_565 ?
I1 Eu1 Eu2 107.802(11) 9_565 11_565 ?
I1 Eu1 Eu2 107.802(11) 9_566 11_565 ?
Eu2 Eu1 Eu2 104.61(3) 1_565 11_565 ?
I4 Eu1 Eu1 111.105(9) 5_455 9_576 ?
I4 Eu1 Eu1 111.105(9) 15 9_576 ?
I1 Eu1 Eu1 170.63(4) 9_565 9_576 ?
I1 Eu1 Eu1 69.365(18) 9_566 9_576 ?
Eu2 Eu1 Eu1 67.181(16) 1_565 9_576 ?
Eu2 Eu1 Eu1 67.181(16) 11_565 9_576 ?
I4 Eu1 Eu1 111.105(9) 5_455 9_575 ?
I4 Eu1 Eu1 111.105(9) 15 9_575 ?
I1 Eu1 Eu1 69.365(18) 9_565 9_575 ?
I1 Eu1 Eu1 170.63(4) 9_566 9_575 ?
Eu2 Eu1 Eu1 67.181(16) 1_565 9_575 ?
Eu2 Eu1 Eu1 67.181(16) 11_565 9_575 ?
Eu1 Eu1 Eu1 101.27(4) 9_576 9_575 ?
I4 Eu1 Eu2 126.906(17) 5_455 3_566 ?
I4 Eu1 Eu2 54.941(12) 15 3_566 ?
I1 Eu1 Eu2 128.159(15) 9_565 3_566 ?
I1 Eu1 Eu2 53.496(10) 9_566 3_566 ?
Eu2 Eu1 Eu2 54.641(12) 1_565 3_566 ?
Eu2 Eu1 Eu2 123.409(15) 11_565 3_566 ?
Eu1 Eu1 Eu2 56.229(8) 9_576 3_566 ?
Eu1 Eu1 Eu2 121.79(2) 9_575 3_566 ?
I4 Eu1 Eu2 54.941(12) 5_455 9_565 ?
I4 Eu1 Eu2 126.906(17) 15 9_565 ?
```

I1 Eu1 Eu2 53.496(10) 9_565 9_565 ?

I1 Eu1 Eu2 128.159(15) 9_566 9_565 ?

Eu2 Eu1 Eu2 123.409(15) 1_565 9_565 ?

Eu2 Eu1 Eu2 54.641(12) 11_565 9_565 ?

Eu1 Eu1 Eu2 121.79(2) 9_576 9_565 ?

Eu1 Eu1 Eu2 56.229(8) 9_575 9_565 ?

Eu2 Eu1 Eu2 177.37(2) 3_566 9_565 ?

I4 Eu1 Eu2 54.941(12) 5_455 9_566 ?

I4 Eu1 Eu2 126.906(17) 15 9_566 ?

I1 Eu1 Eu2 128.159(15) 9_565 9_566 ?

I1 Eu1 Eu2 53.496(10) 9_566 9_566 ?

Eu2 Eu1 Eu2 123.409(15) 1_565 9_566 ?

Eu2 Eu1 Eu2 54.641(12) 11_565 9_566 ?

Eu1 Eu1 Eu2 56.229(8) 9_576 9_566 ?

Eu1 Eu1 Eu2 121.79(2) 9_575 9_566 ?

Eu2 Eu1 Eu2 91.059(13) 3_566 9_566 ?

Eu2 Eu1 Eu2 88.881(13) 9_565 9_566 ?

I4 Eu1 Eu2 126.906(17) 5_455 3_565 ?

I4 Eu1 Eu2 54.941(12) 15 3_565 ?

I1 Eu1 Eu2 53.496(10) 9_565 3_565 ?

I1 Eu1 Eu2 128.159(15) 9_566 3_565 ?

Eu2 Eu1 Eu2 54.641(12) 1_565 3_565 ?

Eu2 Eu1 Eu2 123.409(15) 11_565 3_565 ?

Eu1 Eu1 Eu2 121.79(2) 9_576 3_565 ?

Eu1 Eu1 Eu2 56.229(8) 9_575 3_565 ?

Eu2 Eu1 Eu2 88.881(13) 3_566 3_565 ?

Eu2 Eu1 Eu2 91.059(13) 9_565 3_565 ?

Eu2 Eu1 Eu2 177.37(2) 9_566 3_565 ?

O Eu2 O 78.73(19) . 10_556 ?

O Eu2 I4 86.97(13) . . ?

O Eu2 I4 86.97(13) 10_556 . ?

O Eu2 Eu3 39.63(8) . 15_545 ?

O Eu2 Eu3 39.63(8) 10_556 15_545 ?

I4 Eu2 Eu3 91.10(3) . 15_545 ?

O Eu2 I1 140.06(12) . . ?

O Eu2 I1 140.07(12) 10_556 . ?

I4 Eu2 I1 101.27(3) . . ?

Eu3 Eu2 I1 167.62(3) 15_545 . ?

O Eu2 I4 146.67(16) . 13_556 ?

O Eu2 I4 77.70(4) 10_556 13_556 ?

I4 Eu2 I4 68.605(17) . 13_556 ?

Eu3 Eu2 I4 115.813(17) 15_545 13_556 ?

I1 Eu2 I4 69.579(18) . 13_556 ?

O Eu2 I4 77.70(4) . 13 ?

O Eu2 I4 146.67(16) 10_556 13 ?

I4 Eu2 I4 68.605(17) . 13 ?

Eu3 Eu2 I4 115.813(17) 15_545 13 ?

I1 Eu2 I4 69.579(18) . 13 ?

I4 Eu2 I4 111.51(3) 13_556 13 ?

O Eu2 Eu2 37.38(16) . 3 ?

O Eu2 Eu2 102.58(3) 10_556 3 ?

I4 Eu2 Eu2 116.514(11) . 3 ?

Eu3 Eu2 Eu2 65.603(13) 15_545 3 ?

I1 Eu2 Eu2 108.026(10) . 3 ?

I4 Eu2 Eu2 174.86(2) 13_556 3 ?

I4 Eu2 Eu2 71.120(13) 13 3 ?

O Eu2 Eu2 102.58(3) . 3_556 ?

O Eu2 Eu2 37.38(16) 10_556 3_556 ?

I4 Eu2 Eu2 116.514(11) . 3_556 ?

Eu3 Eu2 Eu2 65.603(13) 15_545 3_556 ?

I1 Eu2 Eu2 108.026(10) . 3_556 ?

I4 Eu2 Eu2 71.120(13) 13_556 3_556 ?

I4 Eu2 Eu2 174.86(2) 13 3_556 ?

Eu2 Eu2 Eu2 105.91(3) 3 3_556 ?

O Eu2 Eu1 100.81(14) . 1_545 ?

O Eu2 Eu1 100.82(14) 10_556 1_545 ?

I4 Eu2 Eu1 169.88(3) . 1_545 ?

Eu3 Eu2 Eu1 99.02(2) 15_545 1_545 ?

I1 Eu2 Eu1 68.60(2) . 1_545 ?

I4 Eu2 Eu1 106.488(17) 13_556 1_545 ?

I4 Eu2 Eu1 106.488(17) 13 1_545 ?

Eu2 Eu2 Eu1 68.385(13) 3 1_545 ?

Eu2 Eu2 Eu1 68.385(13) 3_556 1_545 ?

O Eu2 Eu3 95.99(14) . 13_556 ?

O Eu2 Eu3 31.27(5) 10_556 13_556 ?

I4 Eu2 Eu3 61.881(14) . 13_556 ?

Eu3 Eu2 Eu3 63.578(18) 15_545 13_556 ?

I1 Eu2 Eu3 122.514(15) . 13_556 ?

I4 Eu2 Eu3 52.963(15) 13_556 13_556 ?

I4 Eu2 Eu3 130.37(2) 13 13_556 ?

Eu2 Eu2 Eu3 129.061(18) 3 13_556 ?

Eu2 Eu2 Eu3 54.775(9) 3_556 13_556 ?

Eu1 Eu2 Eu3 122.941(13) 1_545 13_556 ?

O Eu2 Eu3 31.27(5) . 13 ?

O Eu2 Eu3 95.99(14) 10_556 13 ?

I4 Eu2 Eu3 61.881(14) . 13 ?

Eu3 Eu2 Eu3 63.578(18) 15_545 13 ?

I1 Eu2 Eu3 122.514(15) . 13 ?

I4 Eu2 Eu3 130.37(2) 13_556 13 ?

I4 Eu2 Eu3 52.963(15) 13 13 ?

Eu2 Eu2 Eu3 54.775(9) 3 13 ?

Eu2 Eu2 Eu3 129.061(18) 3_556 13 ?

Eu1 Eu2 Eu3 122.941(13) 1_545 13 ?

Eu3 Eu2 Eu3 98.24(2) 13_556 13 ?

O Eu2 H 78(3) . . ?

O Eu2 H 124.1(10) 10_556 . ?

I4 Eu2 H 140.9(9) . . ?

Eu3 Eu2 H 99(2) 15_545 . ?

I1 Eu2 H 71(2) . . ?

I4 Eu2 H 135(3) 13_556 . ?

I4 Eu2 H 73.0(4) 13 . ?

Eu2 Eu2 H 41(3) 3 . ?

Eu2 Eu2 H 102.0(5) 3_556 . ?

Eu1 Eu2 H 37.4(16) 1_545 . ?

Eu3 Eu2 H 154.6(5) 13_556 . ?

Eu3 Eu2 H 89(2) 13 . ?

O Eu3 O 81.36(17) 6 13 ?

O Eu3 I3B 82.2(2) 6 10_556 ?

O Eu3 I3B 109.7(2) 13 10_556 ?

O Eu3 I3B 109.7(2) 6 . ?

O Eu3 I3B 82.2(2) 13 . ?

I3B Eu3 I3B 42.6(4) 10_556 . ?

O Eu3 I3A 99.44(18) 6 . ?

O Eu3 I3A 84.49(18) 13 . ?

I3B Eu3 I3A 33.2(3) 10_556 . ?

I3B Eu3 I3A 11.13(16) . . ?

O Eu3 I3A 84.49(18) 6 10_556 ?

O Eu3 I3A 99.44(18) 13 10_556 ?

I3B Eu3 I3A 11.13(16) 10_556 10_556 ?

I3B Eu3 I3A 33.2(3) . 10_556 ?

I3A Eu3 I3A 23.0(2) . 10_556 ?

O Eu3 I3B 125.5(2) 6 9_565 ?

O Eu3 I3B 80.7(2) 13 9_565 ?

I3B Eu3 I3B 57.05(5) 10_556 9_565 ?

I3B Eu3 I3B 16.8(4) . 9_565 ?

I3A Eu3 I3B 27.9(3) . 9_565 ?

I3A Eu3 I3B 48.95(16) 10_556 9_565 ?

O Eu3 I3B 80.7(2) 6 2_565 ?

O Eu3 I3B 125.5(2) 13 2_565 ?

I3B Eu3 I3B 16.8(4) 10_556 2_565 ?

I3B Eu3 I3B 57.05(5) . 2_565 ?

I3A Eu3 I3B 48.95(16) . 2_565 ?

I3A Eu3 I3B 27.9(3) 10_556 2_565 ?

I3B Eu3 I3B 69.4(3) 9_565 2_565 ?

O Eu3 I2 90.61(17) 6 . ?

O Eu3 I2 90.61(17) 13 . ?

I3B Eu3 I2 22.5(2) 10_556 . ?

I3B Eu3 I2 22.5(2) . . ?

I3A Eu3 I2 11.65(12) . . ?

I3A Eu3 I2 11.65(12) 10_556 . ?

I3B Eu3 I2 38.92(19) 9_565 . ?

I3B Eu3 I2 38.92(19) 2_565 . ?

O Eu3 I4 83.56(16) 6 . ?

O Eu3 I4 83.56(16) 13 . ?

I3B Eu3 I4 158.71(18) 10_556 . ?

I3B Eu3 I4 158.71(18) . . ?

I3A Eu3 I4 167.10(9) . . ?

I3A Eu3 I4 167.10(9) 10_556 . ?

I3B Eu3 I4 143.77(18) 9_565 . ?

I3B Eu3 I4 143.77(18) 2_565 . ?

I2 Eu3 I4 172.30(9) . . ?

O Eu3 I3A 134.23(14) 6 9_565 ?

O Eu3 I3A 79.27(17) 13 9_565 ?

I3B Eu3 I3A 66.36(13) 10_556 9_565 ?

I3B Eu3 I3A 26.7(3) . 9_565 ?

I3A Eu3 I3A 37.8(2) . 9_565 ?

I3A Eu3 I3A 58.70(6) 10_556 9_565 ?

I3B Eu3 I3A 9.95(15) 9_565 9_565 ?

I3B Eu3 I3A 77.7(2) 2_565 9_565 ?

I2 Eu3 I3A 48.85(11) . 9_565 ?

I4 Eu3 I3A 134.11(10) . 9_565 ?

O Eu3 I3A 79.27(17) 6 2_565 ?

O Eu3 I3A 134.23(14) 13 2_565 ?

I3B Eu3 I3A 26.7(3) 10_556 2_565 ?

I3B Eu3 I3A 66.36(13) . 2_565 ?

I3A Eu3 I3A 58.70(6) . 2_565 ?

I3A Eu3 I3A 37.8(2) 10_556 2_565 ?

I3B Eu3 I3A 77.7(2) 9_565 2_565 ?

I3B Eu3 I3A 9.95(15) 2_565 2_565 ?

I2 Eu3 I3A 48.85(11) . 2_565 ?

I4 Eu3 I3A 134.11(10) . 2_565 ?

I3A Eu3 I3A 85.35(17) 9_565 2_565 ?

Eu3 O Eu3 119.7(3) 15_545 13 ?

Eu3 O Eu2 116.49(4) 15_545 3 ?

Eu3 O Eu2 99.41(3) 13 3 ?

Eu3 O Eu2 99.41(3) 15_545 . ?

Eu3 O Eu2 116.49(4) 13 . ?

Eu2 O Eu2 105.2(3) 3 . ?

Eu1 I1 Eu1 120.00(5) 9_565 9_566 ?

Eu1 I1 Eu3 110.925(13) 9_565 11 ?

Eu1 I1 Eu3 110.925(13) 9_566 11 ?

Eu1 I1 Eu3 110.925(13) 9_565 . ?

Eu1 I1 Eu3 110.925(13) 9_566 . ?

Eu3 I1 Eu3 88.83(3) 11 . ?

Eu1 I1 Eu2 75.118(19) 9_565 . ?

Eu1 I1 Eu2 75.118(19) 9_566 . ?

Eu3 I1 Eu2 165.32(3) 11 . ?

Eu3 I1 Eu2 76.492(16) . . ?

Eu1 I1 Eu2 75.118(19) 9_565 11 ?
Eu1 I1 Eu2 75.118(19) 9_566 11 ?
Eu3 I1 Eu2 76.492(16) 11 11 ?
Eu3 I1 Eu2 165.32(3) . 11 ?
Eu2 I1 Eu2 118.19(4) . 11 ?
I3A I2 I3A 156.7(15) 10_556 . ?
I3A I2 I3B 145.0(11) 10_556 . ?
I3A I2 I3B 11.8(7) . . ?
I3A I2 I3B 11.8(7) 10_556 10_556 ?
I3A I2 I3B 145.0(11) . 10_556 ?
I3B I2 I3B 133.2(8) . 10_556 ?
I3A I2 I3B 136.8(9) 10_556 9_565 ?
I3A I2 I3B 19.9(7) . 9_565 ?
I3B I2 I3B 8.2(5) . 9_565 ?
I3B I2 I3B 125.0(4) 10_556 9_565 ?
I3A I2 I3B 19.9(7) 10_556 2_565 ?
I3A I2 I3B 136.8(9) . 2_565 ?
I3B I2 I3B 125.0(4) . 2_565 ?
I3B I2 I3B 8.2(5) 10_556 2_565 ?
I3B I2 I3B 116.9(5) 9_565 2_565 ?
I3A I2 I3A 135.8(8) 10_556 9_565 ?
I3A I2 I3A 20.9(7) . 9_565 ?
I3B I2 I3A 9.1(4) . 9_565 ?
I3B I2 I3A 124.1(5) 10_556 9_565 ?
I3B I2 I3A 1.0(2) 9_565 9_565 ?
I3B I2 I3A 115.9(3) 2_565 9_565 ?
I3A I2 I3A 20.9(7) 10_556 2_565 ?
I3A I2 I3A 135.8(8) . 2_565 ?
I3B I2 I3A 124.1(5) . 2_565 ?
I3B I2 I3A 9.1(4) 10_556 2_565 ?
I3B I2 I3A 115.9(3) 9_565 2_565 ?
I3B I2 I3A 1.0(2) 2_565 2_565 ?
I3A I2 I3A 114.9(2) 9_565 2_565 ?
I3A I2 Eu3 82.5(5) 10_556 . ?
I3A I2 Eu3 82.4(5) . . ?
I3B I2 Eu3 75.0(3) . . ?
I3B I2 Eu3 75.0(3) 10_556 . ?
I3B I2 Eu3 70.06(17) 9_565 . ?
I3B I2 Eu3 70.06(17) 2_565 . ?
I3A I2 Eu3 69.50(10) 9_565 . ?
I3A I2 Eu3 69.50(10) 2_565 . ?
I3A I2 Eu3 82.5(5) 10_556 11 ?
I3A I2 Eu3 82.4(5) . 11 ?
I3B I2 Eu3 75.0(3) . 11 ?
I3B I2 Eu3 75.0(3) 10_556 11 ?
I3B I2 Eu3 70.06(17) 9_565 11 ?
I3B I2 Eu3 70.06(17) 2_565 11 ?

I3A I2 Eu3 69.50(10) 9_565 11 ?

I3A I2 Eu3 69.50(10) 2_565 11 ?

Eu3 I2 Eu3 98.70(17) . 11 ?

I3B I3A I2 155.8(13) . . ?

I3B I3A I3A 144.2(8) . 10_556 ?

I2 I3A I3A 11.6(8) . 10_556 ?

I3B I3A I3B 4.0(10) . 9_565 ?

I2 I3A I3B 151.8(9) . 9_565 ?

I3A I3A I3B 140.1(3) 10_556 9_565 ?

I3B I3A I3B 132.7(11) . 10_556 ?

I2 I3A I3B 23.1(7) . 10_556 ?

I3A I3A I3B 11.5(3) 10_556 10_556 ?

I3B I3A I3B 128.6(3) 9_565 10_556 ?

I3B I3A I3A 2.9(7) . 9_565 ?

I2 I3A I3A 152.9(9) . 9_565 ?

I3A I3A I3A 141.3(2) 10_556 9_565 ?

I3B I3A I3A 1.1(3) 9_565 9_565 ?

I3B I3A I3A 129.8(4) 10_556 9_565 ?

I3B I3A I3B 122.3(7) . 2_565 ?

I2 I3A I3B 33.5(7) . 2_565 ?

I3A I3A I3B 21.9(2) 10_556 2_565 ?

I3B I3A I3B 118.3(4) 9_565 2_565 ?

I3B I3A I3B 10.4(4) 10_556 2_565 ?

I3A I3A I3B 119.4(3) 9_565 2_565 ?

I3B I3A I2 3.3(8) . 9_565 ?

I2 I3A I2 159.1(7) . 9_565 ?

I3A I3A I2 147.47(12) 10_556 9_565 ?

I3B I3A I2 7.3(3) 9_565 9_565 ?

I3B I3A I2 136.0(3) 10_556 9_565 ?

I3A I3A I2 6.2(2) 9_565 9_565 ?

I3B I3A I2 125.6(3) 2_565 9_565 ?

I3B I3A Eu3 78.6(5) . . ?

I2 I3A Eu3 85.9(5) . . ?

I3A I3A Eu3 78.50(12) 10_556 . ?

I3B I3A Eu3 76.08(19) 9_565 . ?

I3B I3A Eu3 71.46(19) 10_556 . ?

I3A I3A Eu3 76.79(17) 9_565 . ?

I3B I3A Eu3 65.54(11) 2_565 . ?

I2 I3A Eu3 80.67(14) 9_565 . ?

I3B I3A Eu3 78.6(5) . 11 ?

I2 I3A Eu3 85.9(5) . 11 ?

I3A I3A Eu3 78.50(11) 10_556 11 ?

I3B I3A Eu3 76.08(19) 9_565 11 ?

I3B I3A Eu3 71.46(19) 10_556 11 ?

I3A I3A Eu3 76.79(17) 9_565 11 ?

I3B I3A Eu3 65.54(11) 2_565 11 ?

I2 I3A Eu3 80.67(14) 9_565 11 ?

Eu3 I3A Eu3 99.53(13) . 11 ?

I3B I3A Eu3 63.6(5) . 9_565 ?

I2 I3A Eu3 128.9(3) . 9_565 ?

I3A I3A Eu3 132.67(8) 10_556 9_565 ?

I3B I3A Eu3 66.1(2) 9_565 9_565 ?

I3B I3A Eu3 134.43(7) 10_556 9_565 ?

I3A I3A Eu3 65.38(18) 9_565 9_565 ?

I3B I3A Eu3 134.11(6) 2_565 9_565 ?

I2 I3A Eu3 61.65(12) 9_565 9_565 ?

Eu3 I3A Eu3 142.2(2) . 9_565 ?

Eu3 I3A Eu3 72.63(6) 11 9_565 ?

I3B I3A Eu3 63.6(5) . 3_565 ?

I2 I3A Eu3 128.9(3) . 3_565 ?

I3A I3A Eu3 132.67(9) 10_556 3_565 ?

I3B I3A Eu3 66.1(2) 9_565 3_565 ?

I3B I3A Eu3 134.43(7) 10_556 3_565 ?

I3A I3A Eu3 65.38(18) 9_565 3_565 ?

I3B I3A Eu3 134.11(6) 2_565 3_565 ?

I2 I3A Eu3 61.65(12) 9_565 3_565 ?

Eu3 I3A Eu3 72.63(6) . 3_565 ?

Eu3 I3A Eu3 142.2(2) 11 3_565 ?

Eu3 I3A Eu3 90.94(14) 9_565 3_565 ?

I3A I3B I3B 173.3(16) . 9_565 ?

I3A I3B I2 12.4(7) . . ?

I3B I3B I2 160.9(12) 9_565 . ?

I3A I3B I3A 176.0(10) . 9_565 ?

I3B I3B I3A 2.7(6) 9_565 9_565 ?

I2 I3B I3A 163.6(6) . 9_565 ?

I3A I3B I3A 24.3(6) . 10_556 ?

I3B I3B I3A 149.0(11) 9_565 10_556 ?

I2 I3B I3A 11.9(3) . 10_556 ?

I3A I3B I3A 151.6(5) 9_565 10_556 ?

I3A I3B I2 175.7(10) . 9_565 ?

I3B I3B I2 11.0(7) 9_565 9_565 ?

I2 I3B I2 171.8(5) . 9_565 ?

I3A I3B I2 8.3(3) 9_565 9_565 ?

I3A I3B I2 159.9(5) 10_556 9_565 ?

I3A I3B I3B 35.8(8) . 10_556 ?

I3B I3B I3B 137.5(9) 9_565 10_556 ?

I2 I3B I3B 23.4(4) . 10_556 ?

I3A I3B I3B 140.1(3) 9_565 10_556 ?

I3A I3B I3B 11.5(3) 10_556 10_556 ?

I2 I3B I3B 148.4(2) 9_565 10_556 ?

I3A I3B I3A 166.0(9) . 2_564 ?

I3B I3B I3A 20.6(8) 9_565 2_564 ?

I2 I3B I3A 178.5(6) . 2_564 ?

I3A I3B I3A 18.0(3) 9_565 2_564 ?

I3A I3B I3A 169.6(4) 10_556 2_564 ?

I2 I3B I3A 9.7(2) 9_565 2_564 ?

I3B I3B I3A 158.1(2) 10_556 2_564 ?

I3A I3B I3B 156.1(10) . 2_564 ?

I3B I3B I3B 30.6(7) 9_565 2_564 ?

I2 I3B I3B 168.5(7) . 2_564 ?

I3A I3B I3B 27.9(3) 9_565 2_564 ?

I3A I3B I3B 179.6(5) 10_556 2_564 ?

I2 I3B I3B 19.6(3) 9_565 2_564 ?

I3B I3B I3B 168.0(3) 10_556 2_564 ?

I3A I3B I3B 9.93(16) 2_564 2_564 ?

I3A I3B I3B 47.8(7) . 2_565 ?

I3B I3B I3B 125.5(11) 9_565 2_565 ?

I2 I3B I3B 35.4(3) . 2_565 ?

I3A I3B I3B 128.2(5) 9_565 2_565 ?

I3A I3B I3B 23.5(2) 10_556 2_565 ?

I2 I3B I3B 136.5(5) 9_565 2_565 ?

I3B I3B I3B 11.9(3) 10_556 2_565 ?

I3A I3B I3B 146.2(5) 2_564 2_565 ?

I3B I3B I3B 156.1(6) 2_564 2_565 ?

I3A I3B Eu3 90.3(5) . . ?

I3B I3B Eu3 85.5(6) 9_565 . ?

I2 I3B Eu3 82.5(3) . . ?

I3A I3B Eu3 87.2(3) 9_565 . ?

I3A I3B Eu3 75.3(2) 10_556 . ?

I2 I3B Eu3 92.4(3) 9_565 . ?

I3B I3B Eu3 68.71(18) 10_556 . ?

I3A I3B Eu3 98.4(3) 2_564 . ?

I3B I3B Eu3 104.4(4) 2_564 . ?

I3B I3B Eu3 62.54(17) 2_565 . ?

I3A I3B Eu3 90.3(5) . 11 ?

I3B I3B Eu3 85.5(6) 9_565 11 ?

I2 I3B Eu3 82.5(3) . 11 ?

I3A I3B Eu3 87.2(3) 9_565 11 ?

I3A I3B Eu3 75.3(2) 10_556 11 ?

I2 I3B Eu3 92.4(3) 9_565 11 ?

I3B I3B Eu3 68.71(18) 10_556 11 ?

I3A I3B Eu3 98.4(3) 2_564 11 ?

I3B I3B Eu3 104.4(4) 2_564 11 ?

I3B I3B Eu3 62.54(17) 2_565 11 ?

Eu3 I3B Eu3 102.3(2) . 11 ?

Eu2 I4 Eu1 166.77(4) . 5_545 ?

Eu2 I4 Eu3 80.76(2) . . ?

Eu1 I4 Eu3 112.47(3) 5_545 . ?

Eu2 I4 Eu2 111.395(17) . 13_556 ?

Eu1 I4 Eu2 75.10(2) 5_545 13_556 ?

Eu3 I4 Eu2 68.630(18) . 13_556 ?

Eu2 I4 Eu2 111.395(17) . 13 ?

Eu1 I4 Eu2 75.10(2) 5_545 13 ?

Eu3 I4 Eu2 68.630(18) . 13 ?

Eu2 I4 Eu2 111.51(3) 13_556 13 ?

_diffrn_measured_fraction_theta_max 1.000

_diffrn_reflns_theta_full 27.88

_diffrn_measured_fraction_theta_full 1.000

_refine_diff_density_max 4.203

_refine_diff_density_min -2.860

_refine_diff_density_rms 0.491

```
_audit_creation_method            SHELXL-97
_chemical_name_systematic
;
 ?
;
_chemical_name_common             ?
_chemical_melting_point           ?
_chemical_formula_moiety          ?
_chemical_formula_sum
 'Eu5 H2 I4 O2'
_chemical_formula_weight          1301.42

loop_
 _atom_type_symbol
 _atom_type_description
 _atom_type_scat_dispersion_real
 _atom_type_scat_dispersion_imag
 _atom_type_scat_source
 'Eu'  'Eu'  -0.1578   3.6682
 'International Tables Vol C Tables 4.2.6.8 and 6.1.1.4'
 'H'  'H'   0.0000   0.0000
 'International Tables Vol C Tables 4.2.6.8 and 6.1.1.4'
 'O'  'O'   0.0106   0.0060
 'International Tables Vol C Tables 4.2.6.8 and 6.1.1.4'
 'I'  'I'  -0.4742   1.8119
 'International Tables Vol C Tables 4.2.6.8 and 6.1.1.4'

_symmetry_cell_setting            orthorhombic
_symmetry_space_group_name_H-M    Cmcm

loop_
 _symmetry_equiv_pos_as_xyz
 'x, y, z'
 '-x, -y, z+1/2'
 'x, -y, -z'
 '-x, y, -z+1/2'
 'x+1/2, y+1/2, z'
 '-x+1/2, -y+1/2, z+1/2'
 'x+1/2, -y+1/2, -z'
 '-x+1/2, y+1/2, -z+1/2'
 '-x, -y, -z'
 'x, y, -z-1/2'
 '-x, y, z'
 'x, -y, z-1/2'
 '-x+1/2, -y+1/2, -z'
 'x+1/2, y+1/2, -z-1/2'
 '-x+1/2, y+1/2, z'
```

'x+1/2, -y+1/2, z-1/2'

_cell_length_a 16.3697(9)
_cell_length_b 13.6954(8)
_cell_length_c 6.0436(4)
_cell_angle_alpha 90.00
_cell_angle_beta 90.00
_cell_angle_gamma 90.00
_cell_volume 1354.91(14)
_cell_formula_units_Z 4
_cell_measurement_temperature 100(2)
_cell_measurement_reflns_used 1695
_cell_measurement_theta_min 0.41
_cell_measurement_theta_max 34.97

_exptl_crystal_description 'lath shaped'
_exptl_crystal_colour 'red'
_exptl_crystal_size_max 0.220
_exptl_crystal_size_mid 0.085
_exptl_crystal_size_min 0.048
_exptl_crystal_density_meas ?
_exptl_crystal_density_diffrn 6.380
_exptl_crystal_density_method 'not measured'
_exptl_crystal_F_000 2180
_exptl_absorpt_coefficient_mu 31.880
_exptl_absorpt_correction_type empirical
_exptl_absorpt_correction_T_min 0.001
_exptl_absorpt_correction_T_max 0.220
_exptl_absorpt_process_details 'SCALEPACK (W. Minor & Z. Otwinowski)'

_exptl_special_details
;
 ?
;

_diffrn_ambient_temperature 100(2)
_diffrn_radiation_wavelength 0.71069
_diffrn_radiation_type MoK\a
_diffrn_radiation_source 'fine-focus sealed tube'
_diffrn_radiation_monochromator graphite
_diffrn_measurement_device_type 'Kappa CCD (Nonius)'
_diffrn_measurement_method 'four circle, CCD'
_diffrn_detector_area_resol_mean ?
_diffrn_standards_number ?
_diffrn_standards_interval_count ?
_diffrn_standards_interval_time ?
_diffrn_standards_decay_% ?

_diffrn_reflns_number 2931
_diffrn_reflns_av_R_equivalents 0.0643
_diffrn_reflns_av_sigmaI/netI 0.0298
_diffrn_reflns_limit_h_min -26
_diffrn_reflns_limit_h_max 26
_diffrn_reflns_limit_k_min -22
_diffrn_reflns_limit_k_max 22
_diffrn_reflns_limit_l_min -9
_diffrn_reflns_limit_l_max 9
_diffrn_reflns_theta_min 1.94
_diffrn_reflns_theta_max 34.96
_reflns_number_total 1645
_reflns_number_gt 1449
_reflns_threshold_expression >2sigma(I)

_computing_data_collection 'Nonius Collect-Software'
_computing_cell_refinement 'Denzo (W. Minor & Z. Otwinowski)'
_computing_data_reduction 'Denzo (W. Minor & Z. Otwinowski)'
_computing_structure_solution 'SHELXS-97 (Sheldrick, 1990)'
_computing_structure_refinement 'SHELXL-97 (Sheldrick, 1997)'
_computing_molecular_graphics ?
_computing_publication_material ?

_refine_special_details
;
Refinement of F^2^ against ALL reflections. The weighted R-factor wR and
goodness of fit S are based on F^2^, conventional R-factors R are based
on F, with F set to zero for negative F^2^. The threshold expression of
F^2^ > 2sigma(F^2^) is used only for calculating R-factors(gt) etc. and is
not relevant to the choice of reflections for refinement. R-factors based
on F^2^ are statistically about twice as large as those based on F, and R-
factors based on ALL data will be even larger.
;

_refine_ls_structure_factor_coef Fsqd
_refine_ls_matrix_type full
_refine_ls_weighting_scheme calc
_refine_ls_weighting_details
'calc w=1/[\s^2^(Fo^2^)+(0.0504P)^2^+34.6527P] where P=(Fo^2^+2Fc^2^)/3'
_atom_sites_solution_primary direct
_atom_sites_solution_secondary difmap
_refine_ls_extinction_method SHELXL
_refine_ls_extinction_coef 0.00087(5)
_refine_ls_extinction_expression
'Fc^*^=kFc[1+0.001xFc^2^\l^3^/sin(2\q)]^-1/4^'
_refine_ls_number_reflns 1645
_refine_ls_number_parameters 46

```
_refine_ls_number_restraints      0
_refine_ls_R_factor_all          0.0415
_refine_ls_R_factor_gt           0.0357
_refine_ls_wR_factor_ref         0.0921
_refine_ls_wR_factor_gt          0.0893
_refine_ls_goodness_of_fit_ref    1.090
_refine_ls_restrained_S_all       1.090
_refine_ls_shift/su_max          0.000
_refine_ls_shift/su_mean         0.000

loop_
 _atom_site_label
 _atom_site_type_symbol
 _atom_site_fract_x
 _atom_site_fract_y
 _atom_site_fract_z
 _atom_site_U_iso_or_equiv
 _atom_site_adp_type
 _atom_site_occupancy
 _atom_site_symmetry_multiplicity
 _atom_site_calc_flag
 _atom_site_refinement_flags
 _atom_site_disorder_assembly
 _atom_site_disorder_group
Eu1 Eu 0.0000 0.90892(4) 0.2500 0.00730(13) Uani 1 4 d S . .
Eu2 Eu 0.18814(2) 0.08380(3) 0.2500 0.00497(11) Uani 1 2 d S . .
Eu3 Eu 0.15203(2) 0.40322(3) 0.2500 0.00665(11) Uani 1 2 d S . .
O O 0.2762(3) 0.0000 0.0000 0.0076(10) Uani 1 2 d S . .
H H 0.090(7) 0.0000 0.0000 0.019 Uiso 1 2 d S . .
I1 I 0.0000 0.21783(6) 0.2500 0.00813(15) Uani 1 4 d S . .
I2 I 0.0000 0.5589(4) 0.2500 0.017(2) Uani 0.232(15) 4 d SP . .
I3 I 0.0000 0.5436(4) 0.1192(15) 0.047(2) Uani 0.377(9) 2 d SP . .
I4 I 0.32809(3) 0.26778(4) 0.2500 0.00756(12) Uani 1 2 d S . .

loop_
 _atom_site_aniso_label
 _atom_site_aniso_U_11
 _atom_site_aniso_U_22
 _atom_site_aniso_U_33
 _atom_site_aniso_U_23
 _atom_site_aniso_U_13
 _atom_site_aniso_U_12
Eu1 0.0062(2) 0.0025(2) 0.0133(3) 0.000 0.000 0.000
Eu2 0.00428(16) 0.00080(16) 0.00982(19) 0.000 0.000 0.00070(12)
Eu3 0.00314(17) 0.00306(18) 0.0138(2) 0.000 0.000 -0.00106(11)
O 0.007(2) 0.004(2) 0.012(3) -0.001(2) 0.000 0.000
I1 0.0045(3) 0.0100(3) 0.0099(3) 0.000 0.000 0.000
```

I2 0.0130(18) 0.018(2) 0.020(5) 0.000 0.000 0.000
I3 0.0142(9) 0.0391(17) 0.089(5) 0.042(3) 0.000 0.000
I4 0.0049(2) 0.0058(2) 0.0120(2) 0.000 0.000 -0.00293(15)

_geom_special_details
;
 All esds (except the esd in the dihedral angle between two l.s. planes)
 are estimated using the full covariance matrix. The cell esds are taken
 into account individually in the estimation of esds in distances, angles
 and torsion angles; correlations between esds in cell parameters are only
 used when they are defined by crystal symmetry. An approximate (isotropic)
 treatment of cell esds is used for estimating esds involving l.s. planes.
;

loop_
 _geom_bond_atom_site_label_1
 _geom_bond_atom_site_label_2
 _geom_bond_distance
 _geom_bond_site_symmetry_2
 _geom_bond_publ_flag
Eu1 I4 3.4141(6) 5_455 ?
Eu1 I4 3.4141(6) 15 ?
Eu1 I1 3.4849(5) 9_565 ?
Eu1 I1 3.4849(5) 9_566 ?
Eu1 Eu2 3.9015(6) 1_565 ?
Eu1 Eu2 3.9015(6) 11_565 ?
Eu1 Eu1 3.9187(7) 9_576 ?
Eu1 Eu1 3.9187(7) 9_575 ?
Eu1 Eu2 4.3158(3) 3_566 ?
Eu1 Eu2 4.3158(3) 9_565 ?
Eu1 Eu2 4.3158(3) 9_566 ?
Eu1 Eu2 4.3158(3) 3_565 ?
Eu2 O 2.383(3) . ?
Eu2 O 2.383(3) 10_556 ?
Eu2 I4 3.4054(6) . ?
Eu2 I1 3.5853(6) . ?
Eu2 Eu3 3.6002(6) 15_545 ?
Eu2 I4 3.6516(4) 13_556 ?
Eu2 I4 3.6516(4) 13 ?
Eu2 Eu2 3.7946(5) 3 ?
Eu2 Eu2 3.7946(5) 3_556 ?
Eu2 Eu1 3.9015(6) 1_545 ?
Eu2 Eu3 4.0010(4) 13_556 ?
Eu2 Eu3 4.0010(4) 13 ?
Eu2 H 2.49(7) . ?
Eu3 O 2.328(3) 6 ?
Eu3 O 2.328(3) 13 ?

Eu3 I3 3.2426(16) 10_556 ?
Eu3 I3 3.2426(16) . ?
Eu3 I2 3.277(3) . ?
Eu3 I3 3.421(5) 9_565 ?
Eu3 I3 3.421(5) 2_565 ?
Eu3 I4 3.4273(7) . ?
Eu3 I1 3.5554(7) . ?
Eu3 Eu2 3.6001(6) 15 ?
Eu3 Eu2 4.0010(4) 13_556 ?
Eu3 Eu2 4.0010(4) 13 ?
O Eu3 2.328(3) 15_545 ?
O Eu3 2.328(3) 13 ?
O Eu2 2.383(3) 3 ?
I1 Eu1 3.4848(5) 9_565 ?
I1 Eu1 3.4848(5) 9_566 ?
I1 Eu3 3.5553(7) 11 ?
I1 Eu2 3.5854(6) 11 ?
I2 I3 0.818(10) 10_556 ?
I2 I3 0.818(10) . ?
I2 I3 2.636(9) 9_565 ?
I2 I3 2.636(9) 2_565 ?
I2 Eu3 3.277(3) 11 ?
I3 I3 1.581(18) 10_556 ?
I3 I3 1.87(2) 9_565 ?
I3 I2 2.636(9) 9_565 ?
I3 Eu3 3.2426(16) 11 ?
I3 Eu3 3.421(5) 9_565 ?
I3 Eu3 3.421(5) 3_565 ?
I4 Eu1 3.4141(6) 5_545 ?
I4 Eu2 3.6516(4) 13_556 ?
I4 Eu2 3.6516(4) 13 ?

loop_
_geom_angle_atom_site_label_1
_geom_angle_atom_site_label_2
_geom_angle_atom_site_label_3
_geom_angle
_geom_angle_site_symmetry_1
_geom_angle_site_symmetry_3
_geom_angle_publ_flag
I4 Eu1 I4 111.03(2) 5_455 15 ?
I4 Eu1 I1 73.620(11) 5_455 9_565 ?
I4 Eu1 I1 73.620(11) 15 9_565 ?
I4 Eu1 I1 73.620(11) 5_455 9_566 ?
I4 Eu1 I1 73.620(11) 15 9_566 ?
I1 Eu1 I1 120.25(3) 9_565 9_566 ?
I4 Eu1 Eu2 176.613(19) 5_455 1_565 ?

I4 Eu1 Eu2 72.354(12) 15 1_565 ?
I1 Eu1 Eu2 107.805(7) 9_565 1_565 ?
I1 Eu1 Eu2 107.805(7) 9_566 1_565 ?
I4 Eu1 Eu2 72.354(12) 5_455 11_565 ?
I4 Eu1 Eu2 176.613(19) 15 11_565 ?
I1 Eu1 Eu2 107.805(7) 9_565 11_565 ?
I1 Eu1 Eu2 107.805(7) 9_566 11_565 ?
Eu2 Eu1 Eu2 104.259(19) 1_565 11_565 ?
I4 Eu1 Eu1 111.129(6) 5_455 9_576 ?
I4 Eu1 Eu1 111.129(6) 15 9_576 ?
I1 Eu1 Eu1 170.33(2) 9_565 9_576 ?
I1 Eu1 Eu1 69.419(13) 9_566 9_576 ?
Eu2 Eu1 Eu1 66.993(11) 1_565 9_576 ?
Eu2 Eu1 Eu1 66.993(11) 11_565 9_576 ?
I4 Eu1 Eu1 111.129(6) 5_455 9_575 ?
I4 Eu1 Eu1 111.129(6) 15 9_575 ?
I1 Eu1 Eu1 69.419(13) 9_565 9_575 ?
I1 Eu1 Eu1 170.33(2) 9_566 9_575 ?
Eu2 Eu1 Eu1 66.993(11) 1_565 9_575 ?
Eu2 Eu1 Eu1 66.993(11) 11_565 9_575 ?
Eu1 Eu1 Eu1 100.91(3) 9_576 9_575 ?
I4 Eu1 Eu2 126.964(11) 5_455 3_566 ?
I4 Eu1 Eu2 54.892(7) 15 3_566 ?
I1 Eu1 Eu2 128.218(10) 9_565 3_566 ?
I1 Eu1 Eu2 53.444(7) 9_566 3_566 ?
Eu2 Eu1 Eu2 54.724(8) 1_565 3_566 ?
Eu2 Eu1 Eu2 123.307(10) 11_565 3_566 ?
Eu1 Eu1 Eu2 56.314(5) 9_576 3_566 ?
Eu1 Eu1 Eu2 121.681(13) 9_575 3_566 ?
I4 Eu1 Eu2 54.892(7) 5_455 9_565 ?
I4 Eu1 Eu2 126.964(11) 15 9_565 ?
I1 Eu1 Eu2 53.444(7) 9_565 9_565 ?
I1 Eu1 Eu2 128.218(10) 9_566 9_565 ?
Eu2 Eu1 Eu2 123.307(10) 1_565 9_565 ?
Eu2 Eu1 Eu2 54.724(8) 11_565 9_565 ?
Eu1 Eu1 Eu2 121.681(13) 9_576 9_565 ?
Eu1 Eu1 Eu2 56.314(5) 9_575 9_565 ?
Eu2 Eu1 Eu2 177.349(17) 3_566 9_565 ?
I4 Eu1 Eu2 54.892(7) 5_455 9_566 ?
I4 Eu1 Eu2 126.964(11) 15 9_566 ?
I1 Eu1 Eu2 128.218(10) 9_565 9_566 ?
I1 Eu1 Eu2 53.444(7) 9_566 9_566 ?
Eu2 Eu1 Eu2 123.307(10) 1_565 9_566 ?
Eu2 Eu1 Eu2 54.724(8) 11_565 9_566 ?
Eu1 Eu1 Eu2 56.314(5) 9_576 9_566 ?
Eu1 Eu1 Eu2 121.681(13) 9_575 9_566 ?
Eu2 Eu1 Eu2 91.058(9) 3_566 9_566 ?

Eu2 Eu1 Eu2 88.880(9) 9_565 9_566 ?

I4 Eu1 Eu2 126.964(11) 5_455 3_565 ?

I4 Eu1 Eu2 54.892(7) 15 3_565 ?

I1 Eu1 Eu2 53.444(7) 9_565 3_565 ?

I1 Eu1 Eu2 128.218(10) 9_566 3_565 ?

Eu2 Eu1 Eu2 54.724(8) 1_565 3_565 ?

Eu2 Eu1 Eu2 123.307(10) 11_565 3_565 ?

Eu1 Eu1 Eu2 121.681(13) 9_576 3_565 ?

Eu1 Eu1 Eu2 56.314(5) 9_575 3_565 ?

Eu2 Eu1 Eu2 88.880(9) 3_566 3_565 ?

Eu2 Eu1 Eu2 91.058(9) 9_565 3_565 ?

Eu2 Eu1 Eu2 177.349(17) 9_566 3_565 ?

O Eu2 O 78.69(13) . 10_556 ?

O Eu2 I4 87.09(9) . . ?

O Eu2 I4 87.09(9) 10_556 . ?

O Eu2 I1 140.03(8) . . ?

O Eu2 I1 140.03(8) 10_556 . ?

I4 Eu2 I1 101.481(17) . . ?

O Eu2 Eu3 39.60(5) . 15_545 ?

O Eu2 Eu3 39.60(5) 10_556 15_545 ?

I4 Eu2 Eu3 91.110(15) . 15_545 ?

I1 Eu2 Eu3 167.409(18) . 15_545 ?

O Eu2 I4 146.79(11) . 13_556 ?

O Eu2 I4 77.73(3) 10_556 13_556 ?

I4 Eu2 I4 68.718(11) . 13_556 ?

I1 Eu2 I4 69.666(11) . 13_556 ?

Eu3 Eu2 I4 115.802(11) 15_545 13_556 ?

O Eu2 I4 77.73(3) . 13 ?

O Eu2 I4 146.79(11) 10_556 13 ?

I4 Eu2 I4 68.718(11) . 13 ?

I1 Eu2 I4 69.666(11) . 13 ?

Eu3 Eu2 I4 115.802(11) 15_545 13 ?

I4 Eu2 I4 111.692(19) 13_556 13 ?

O Eu2 Eu2 37.24(11) . 3 ?

O Eu2 Eu2 102.33(2) 10_556 3 ?

I4 Eu2 Eu2 116.585(7) . 3 ?

I1 Eu2 Eu2 108.040(6) . 3 ?

Eu3 Eu2 Eu2 65.450(9) 15_545 3 ?

I4 Eu2 Eu2 174.690(13) 13_556 3 ?

I4 Eu2 Eu2 71.199(9) 13 3 ?

O Eu2 Eu2 102.33(2) . 3_556 ?

O Eu2 Eu2 37.24(11) 10_556 3_556 ?

I4 Eu2 Eu2 116.585(7) . 3_556 ?

I1 Eu2 Eu2 108.040(6) . 3_556 ?

Eu3 Eu2 Eu2 65.450(9) 15_545 3_556 ?

I4 Eu2 Eu2 71.199(9) 13_556 3_556 ?

I4 Eu2 Eu2 174.690(13) 13 3_556 ?

Eu2 Eu2 Eu2 105.563(19) 3 3_556 ?

O Eu2 Eu1 100.49(9) . 1_545 ?

O Eu2 Eu1 100.49(9) 10_556 1_545 ?

I4 Eu2 Eu1 170.148(17) . 1_545 ?

I1 Eu2 Eu1 68.668(15) . 1_545 ?

Eu3 Eu2 Eu1 98.742(14) 15_545 1_545 ?

I4 Eu2 Eu1 106.519(11) 13_556 1_545 ?

I4 Eu2 Eu1 106.518(11) 13 1_545 ?

Eu2 Eu2 Eu1 68.204(9) 3 1_545 ?

Eu2 Eu2 Eu1 68.204(9) 3_556 1_545 ?

O Eu2 Eu3 96.00(10) . 13_556 ?

O Eu2 Eu3 31.45(4) 10_556 13_556 ?

I4 Eu2 Eu3 61.785(9) . 13_556 ?

I1 Eu2 Eu3 122.613(9) . 13_556 ?

Eu3 Eu2 Eu3 63.596(11) 15_545 13_556 ?

I4 Eu2 Eu3 52.974(10) 13_556 13_556 ?

I4 Eu2 Eu3 130.378(14) 13 13_556 ?

Eu2 Eu2 Eu3 128.926(11) 3 13_556 ?

Eu2 Eu2 Eu3 54.931(7) 3_556 13_556 ?

Eu1 Eu2 Eu3 122.921(9) 1_545 13_556 ?

O Eu2 Eu3 31.45(4) . 13 ?

O Eu2 Eu3 96.00(10) 10_556 13 ?

I4 Eu2 Eu3 61.785(9) . 13 ?

I1 Eu2 Eu3 122.613(9) . 13 ?

Eu3 Eu2 Eu3 63.596(11) 15_545 13 ?

I4 Eu2 Eu3 130.378(14) 13_556 13 ?

I4 Eu2 Eu3 52.974(10) 13 13 ?

Eu2 Eu2 Eu3 54.931(7) 3 13 ?

Eu2 Eu2 Eu3 128.926(11) 3_556 13 ?

Eu1 Eu2 Eu3 122.921(9) 1_545 13 ?

Eu3 Eu2 Eu3 98.095(13) 13_556 13 ?

O Eu2 H 77(2) . . ?

O Eu2 H 123.6(8) 10_556 . ?

I4 Eu2 H 140.9(7) . . ?

I1 Eu2 H 71.4(18) . . ?

Eu3 Eu2 H 98.8(17) 15_545 . ?

I4 Eu2 H 135(2) 13_556 . ?

I4 Eu2 H 73.0(3) 13 . ?

Eu2 Eu2 H 40(2) 3 . ?

Eu2 Eu2 H 101.8(4) 3_556 . ?

Eu1 Eu2 H 37.5(12) 1_545 . ?

Eu3 Eu2 H 154.4(4) 13_556 . ?

Eu3 Eu2 H 89.1(17) 13 . ?

O Eu3 O 80.95(12) 6 13 ?

O Eu3 I3 83.76(13) 6 10_556 ?

O Eu3 I3 101.99(18) 13 10_556 ?

O Eu3 I3 101.99(18) 6 . ?

O Eu3 I3 83.76(13) 13 . ?

I3 Eu3 I3 28.2(3) 10_556 . ?

O Eu3 I2 90.72(11) 6 . ?

O Eu3 I2 90.72(11) 13 . ?

I3 Eu3 I2 14.40(18) 10_556 . ?

I3 Eu3 I2 14.40(18) . . ?

O Eu3 I3 132.00(14) 6 9_565 ?

O Eu3 I3 79.77(12) 13 9_565 ?

I3 Eu3 I3 58.29(3) 10_556 9_565 ?

I3 Eu3 I3 32.5(3) . 9_565 ?

I2 Eu3 I3 46.29(14) . 9_565 ?

O Eu3 I3 79.77(12) 6 2_565 ?

O Eu3 I3 132.00(14) 13 2_565 ?

I3 Eu3 I3 32.5(3) 10_556 2_565 ?

I3 Eu3 I3 58.29(3) . 2_565 ?

I2 Eu3 I3 46.29(14) . 2_565 ?

I3 Eu3 I3 81.4(3) 9_565 2_565 ?

O Eu3 I4 83.34(11) 6 . ?

O Eu3 I4 83.34(11) 13 . ?

I3 Eu3 I4 165.08(14) 10_556 . ?

I3 Eu3 I4 165.08(14) . . ?

I2 Eu3 I4 172.18(7) . . ?

I3 Eu3 I4 136.63(15) 9_565 . ?

I3 Eu3 I4 136.63(15) 2_565 . ?

O Eu3 I1 139.44(7) 6 . ?

O Eu3 I1 139.44(7) 13 . ?

I3 Eu3 I1 83.46(7) 10_556 . ?

I3 Eu3 I1 83.46(6) . . ?

I2 Eu3 I1 86.16(7) . . ?

I3 Eu3 I1 69.07(4) 9_565 . ?

I3 Eu3 I1 69.07(4) 2_565 . ?

I4 Eu3 I1 101.658(16) . . ?

O Eu3 Eu2 40.74(7) 6 15 ?

O Eu3 Eu2 40.74(7) 13 15 ?

I3 Eu3 Eu2 98.66(6) 10_556 15 ?

I3 Eu3 Eu2 98.66(6) . 15 ?

I2 Eu3 Eu2 96.02(7) . 15 ?

I3 Eu3 Eu2 112.49(4) 9_565 15 ?

I3 Eu3 Eu2 112.49(4) 2_565 15 ?

I4 Eu3 Eu2 76.156(14) . 15 ?

I1 Eu3 Eu2 177.814(16) . 15 ?

O Eu3 Eu2 32.29(6) 6 13_556 ?

O Eu3 Eu2 97.77(10) 13 13_556 ?

I3 Eu3 Eu2 106.94(12) 10_556 13_556 ?

I3 Eu3 Eu2 131.27(17) . 13_556 ?

I2 Eu3 Eu2 117.88(4) . 13_556 ?

I3 Eu3 Eu2 163.51(14) 9_565 13_556 ?

I3 Eu3 Eu2 88.49(11) 2_565 13_556 ?

I4 Eu3 Eu2 58.278(9) . 13_556 ?

I1 Eu3 Eu2 119.306(9) . 13_556 ?

Eu2 Eu3 Eu2 59.619(10) 15 13_556 ?

O Eu3 Eu2 97.77(10) 6 13 ?

O Eu3 Eu2 32.29(6) 13 13 ?

I3 Eu3 Eu2 131.27(17) 10_556 13 ?

I3 Eu3 Eu2 106.94(12) . 13 ?

I2 Eu3 Eu2 117.88(4) . 13 ?

I3 Eu3 Eu2 88.49(11) 9_565 13 ?

I3 Eu3 Eu2 163.51(14) 2_565 13 ?

I4 Eu3 Eu2 58.278(9) . 13 ?

I1 Eu3 Eu2 119.306(9) . 13 ?

Eu2 Eu3 Eu2 59.619(10) 15 13 ?

Eu2 Eu3 Eu2 98.097(13) 13_556 13 ?

Eu3 O Eu3 119.4(2) 15_545 13 ?

Eu3 O Eu2 116.27(3) 15_545 3 ?

Eu3 O Eu2 99.67(2) 13 3 ?

Eu3 O Eu2 99.67(2) 15_545 . ?

Eu3 O Eu2 116.27(3) 13 . ?

Eu2 O Eu2 105.5(2) 3 . ?

Eu1 I1 Eu1 120.25(3) 9_565 9_566 ?

Eu1 I1 Eu3 110.837(8) 9_565 11 ?

Eu1 I1 Eu3 110.837(8) 9_566 11 ?

Eu1 I1 Eu3 110.837(8) 9_565 . ?

Eu1 I1 Eu3 110.837(8) 9_566 . ?

Eu3 I1 Eu3 88.85(2) 11 . ?

Eu1 I1 Eu2 75.226(12) 9_565 . ?

Eu1 I1 Eu2 75.226(12) 9_566 . ?

Eu3 I1 Eu2 165.22(2) 11 . ?

Eu3 I1 Eu2 76.369(10) . . ?

Eu1 I1 Eu2 75.226(12) 9_565 11 ?

Eu1 I1 Eu2 75.226(12) 9_566 11 ?

Eu3 I1 Eu2 76.370(10) 11 11 ?

Eu3 I1 Eu2 165.22(2) . 11 ?

Eu2 I1 Eu2 118.41(3) . 11 ?

I3 I2 I3 150.3(10) 10_556 . ?

I3 I2 I3 133.0(6) 10_556 9_565 ?

I3 I2 I3 17.3(4) . 9_565 ?

I3 I2 I3 17.3(4) 10_556 2_565 ?

I3 I2 I3 133.0(6) . 2_565 ?

I3 I2 I3 115.6(2) 9_565 2_565 ?

I3 I2 Eu3 80.4(3) 10_556 11 ?

I3 I2 Eu3 80.4(3) . 11 ?

I3 I2 Eu3 69.73(9) 9_565 11 ?

I3 I2 Eu3 69.73(9) 2_565 11 ?

I3 I2 Eu3 80.4(3) 10_556 . ?

I3 I2 Eu3 80.4(3) . . ?

I3 I2 Eu3 69.73(9) 9_565 . ?

I3 I2 Eu3 69.73(9) 2_565 . ?

Eu3 I2 Eu3 98.82(14) 11 . ?

I2 I3 I3 14.9(5) . 10_556 ?

I2 I3 I3 155.2(5) . 9_565 ?

I3 I3 I3 140.3(2) 10_556 9_565 ?

I2 I3 I2 162.7(4) . 9_565 ?

I3 I3 I2 147.82(11) 10_556 9_565 ?

I3 I3 I2 7.47(16) 9_565 9_565 ?

I2 I3 Eu3 85.2(3) . 11 ?

I3 I3 Eu3 75.89(17) 10_556 11 ?

I3 I3 Eu3 79.01(12) 9_565 11 ?

I2 I3 Eu3 83.72(15) 9_565 11 ?

I2 I3 Eu3 85.2(3) . . ?

I3 I3 Eu3 75.89(17) 10_556 . ?

I3 I3 Eu3 79.01(12) 9_565 . ?

I2 I3 Eu3 83.72(15) 9_565 . ?

Eu3 I3 Eu3 100.26(7) 11 . ?

I2 I3 Eu3 125.2(3) . 9_565 ?

I3 I3 Eu3 130.71(13) 10_556 9_565 ?

I3 I3 Eu3 68.5(2) 9_565 9_565 ?

I2 I3 Eu3 63.98(19) 9_565 9_565 ?

Eu3 I3 Eu3 74.15(7) 11 9_565 ?

Eu3 I3 Eu3 147.5(3) . 9_565 ?

I2 I3 Eu3 125.2(3) . 3_565 ?

I3 I3 Eu3 130.71(13) 10_556 3_565 ?

I3 I3 Eu3 68.5(2) 9_565 3_565 ?

I2 I3 Eu3 63.98(19) 9_565 3_565 ?

Eu3 I3 Eu3 147.5(3) 11 3_565 ?

Eu3 I3 Eu3 74.15(7) . 3_565 ?

Eu3 I3 Eu3 93.35(18) 9_565 3_565 ?

Eu2 I4 Eu1 166.76(2) . 5_545 ?

Eu2 I4 Eu3 80.492(15) . . ?

Eu1 I4 Eu3 112.749(19) 5_545 . ?

Eu2 I4 Eu2 111.282(11) . 13_556 ?

Eu1 I4 Eu2 75.213(12) 5_545 13_556 ?

Eu3 I4 Eu2 68.748(11) . 13_556 ?

Eu2 I4 Eu2 111.282(11) . 13 ?

Eu1 I4 Eu2 75.213(12) 5_545 13 ?

Eu3 I4 Eu2 68.748(11) . 13 ?

Eu2 I4 Eu2 111.691(19) 13_556 13 ?

_diffrn_measured_fraction_theta_max 0.999

_diffrn_reflns_theta_full 34.96

_diffrn_measured_fraction_theta_full 0.999

_refine_diff_density_max 4.112

_refine_diff_density_min	-3.953
_refine_diff_density_rms	0.594
